# Unveiling the antifungal and antibiofilm potential of green synthesized silver nanoparticles from leaf extract of *Selaginella bryopteris*

**DOI:** 10.1038/s41598-025-97201-1

**Published:** 2025-09-30

**Authors:** Khushbu Wadhwa, Neha Kapoor, Mohd. Tariq, Hardeep Kaur

**Affiliations:** 1 Fungal Biology Laboratory, Department of Zoology, Ramjas College, University of Delhi, Delhi, India; 2 Chemical Biology Laboratory, Department of Chemistry, Hindu College, University of Delhi, Delhi, India; 3 Department of Life Sciences, Parul Institute of Applied Sciences, Parul University, Vadodara, Gujarat 391760 India; 4 Department of Biotechnology, Graphic Era (Deemed to be University), Dehradun, Uttarakhand 248002 India

**Keywords:** *Selaginella bryopteris*, Green-synthesis, Silver nanoparticles, *Candida*, Antifungal, Biofilm, Chemical biology, Molecular biology

## Abstract

The emergence of highly drug-resistant fungal strains is the major concern in health care sector. There is an urgent need to develop novel and potent antifungal drugs with minimal side effects to encounter invasive fungal infections. In this study, we have green-synthesized silver nanoparticles (AgNPs) by using leaf extract of *Selaginella bryopteris* and checked their antifungal activity against different *Candida* spp. The optimization of parameters involved in the synthesis of AgNPs includes pH, temperature, concentration of silver nitrate, reaction time. The synthesis of NPs was investigated by the UV–Vis spectrophotometric analysis. The physicochemical properties of AgNPs were further analysed by FESEM, TEM, DLS, zeta potential, FTIR and XRD studies. AgNPs were found to be spherical in shape with an average size of 35 nm and were monodispersed in nature without any agglomeration. The results of antifungal susceptibility testing (AFST) and growth curve kinetics revealed that AgNPs displayed significant anticandidal activity with MIC and MFC values of 0.003 and 0.006 ng/mL respectively. Treatment of *Candida* spp. with AgNPs leads to damage in fungal cell wall, cell membrane along with disruption of mitochondrial enzyme activity and release of nuclear content. The green-synthesized AgNPs not only caused damage and destruction to the morphology of *Candida* but also affected the ergosterol biosynthetic pathway. The green-synthesized AgNPs were also found to exhibit antibiofilm activity against *Candida* spp. which was assessed by crystal violet assay and SEM analysis confirming biofilm reduction by 80–82% as compared to control.

## Introduction

The unique properties of nanomaterial have drawn more attention due to their extensive use in drug delivery, diagnostics, imaging, probing, gene insertion, manufacturing of artificial implant and tissue engineering^1^. Recent breakthrough in the field of nanotechnology indicates that nanomaterials, play pivotal role in biological, pharmaceutical and biomedical fields^2^. The metal-based nanoparticles (NPs) exhibit unique properties like the large surface area to volume ratio, high surface reaction activity, high mechanical and thermal stability which enables them to penetrate, adhere and invade microbial cell envelopes^3^. In this study, we have synthesized AgNPs by ‘green-synthesis’ approach. Both chemical and physical methods have also been adopted for the synthesis of NPs, but these methods exhibit negative impact on environment as they make use of hazardous chemicals and require complex route for the synthesis^4^^,^^5^. On the other hand, green-synthesized NPs are non-toxic in nature, environmentally friendly, biocompatible and commercially feasible. Till date, plant extract, algae, fungi, bacteria, yeast and biopolymers have been used for the green synthesis of NPs. In addition to AgNPs, the biosynthesis of different types of metallic NPs has also gained prominence due to their unique physicochemical properties such as zinc oxide^6^, zirconium^7^, tellurium^8^ and selenium NPs^9^. The toxicity of NPs is mainly dependent on the size, shape, coating and stabilizing agents used in the synthesis process. The biogenic or green synthesized AgNPs are therefore considered more biocompatible as compared to physically and chemically synthesized AgNPs^10^, due to the involvement of plant derived secondary metabolites that act as reducing and stabilizing agents. Analysis of various studies has revealed that the approach of synthesizing AgNPs by ecofriendly methods from different parts of plant extracts and using them as potent antifungal agents is one of the best innovative strategies to combat invasive fungal infections^11,12^. In one of our studies, we had synthesized the AgNPs by using the leaf extract of *Selaginella bryopteris* and reported its antibacterial activity against human pathogenic bacteria^13^. *Selaginella bryopteris* is known as lycophyte ‘resurrection plant’ due to its immense potential of becoming metabolically active by adding water to its dried bare roots^14^. The leaves of the plant incurve during desiccation and recover on availability of moisture. This herb plant contains different classes of phytocompounds or secondary metabolites such as alkaloids, phenols (flavonoids, tannins, saponins) and terpenoids (triterpene, steroids)^15^. The phytocompounds isolated from *Selaginella,* exhibit antimicrobial^13^, antioxidant^16^, anticancer^15^, anti-allergic^17^, antiplasmodial and leishmanicidal activities^18^. In addition to this, study by Yassin et al.^19^ has also reported the anticandidal activity of n-hexane, diethyl-ether, ethyl-acetate, methanolic extract of *Mentha longifolia* (wild mint) against *C. tropicalis*, *C. albicans* and *C. glabrata*. *Syzygium aromaticum* (clove) has also been tested for its antifungal potency against *C. albicans*, *C. glabrata* and *C. tropicalis* causing vulvovaginal candidiasis (VVC)^20^. The protective and disease preventing effects of phytocompounds is therefore well established. However, the antimicrobial potency of NPs derived using these extracts far exceeds the antimicrobial potency of the extracts when used alone as was established in our previous study and this study as well^21,22^.

*Candida* is defined as an opportunistic fungal pathogen, responsible for mucosal, superficial, cutaneous, and blood stream infections in human. Though *C. albicans* is responsible for 50% of candidiasis in human, the other *non-albicans Candida* (NAC) spp. including *C. parapsilosis*, *C. glabrata*, *C. krusei*, *C. tropicalis* are also responsible for high morbidity and mortality in humans^23^. To treat fungal infections, three classes of antifungal drugs are available including Azoles (fluconazole (FLU), itraconazole (ITC), voriconazole (VRC)), Echinocandins (caspofungin (CFG), micafungin (MFG), anidulafungin (AFG)); and Polyenes (amphotericin B (AmB), nystatin)^24^, but all these drugs exhibit various side effects such as nausea, vomiting, gastrointestinal problems and hepatotoxicity^25^. The situation has become grave with the emergence of multi drug resistant fungal species due to indiscriminate use of these antifungal drugs. There is also an unprecedented increase in nosocomial fungal infections such as urinary tract infections and systemic infections in the recent years. Thus, it requires an immediate attention to develop novel alternate antifungal drugs.

AgNPs are generally used as topical ointments and in dressings for the effective treatment of wounds and for infection control^26^. For example, the United States Food and Drug Administration (USFDA) has authorised the use of silver nanocrystal-based dressings such as, KerraContact Ag and Acticoat Flex 7 for burn treatment^27–29^. The treatment of *Candida* spp. with AgNPs has been found to cause fungal cell wall and cytoplasmic membrane disruption, an increase in membrane permeability, and interference with cellular biomolecules that can further lead to cellular dysfunction and fungal cell death^30,31^.

The primary objective of this study is to synthesize AgNPs using the leaf extract of *S. bryopteris* that have greater biocompatibility in comparison to those synthesized from physical and chemical methods and evaluate their antifungal activity against *Candida* spp. especially against the multidrug resistant strains with an aim to develop effective antifungal drug.

## Materials and methods

### Chemicals and materials

All chemicals used in this study were of analytical grade and were purchased from Sigma and Merck (St. Louis, MO, USA). The medically important antifungal drug, FLU and AmB were purchased from Hi-media (India). Silver nitrate (AgNO_3_) was obtained from Sigma.

### Collection and preparation of leaf extract

For the synthesis of AgNPs, *Selaginella bryopteris* was purchased from the local market of Delhi as it is a popular herb used in Ayurvedic medicines. The herbarium of *S. bryopteris* was duly verified by the plant taxonomist at Department of Botany, University of Delhi and submitted with Herbarium No. DUH15970. The herb was washed thoroughly with distilled water and, then left to air dry for 1–2 days. The leaves were then crushed or grounded to get a fine powder. The obtained powder was stored at 4°C. 2.5 g of leaf powder was added to 50 mL of distilled water and kept on a magnetic stirrer at room temperature (RT) for 24 h. Solution was then filtered with Whatman filter paper number-1^32^. The aqueous leaf extract was stored at 4°C and used as a reducing and stabilizing agent for the green synthesis of AgNPs.

### Green synthesis of AgNPs

For the preparation of solvent, 45 mL of 1mM (0.001M) aqueous solution of AgNO_3_ was prepared and stored in an amber bottle to prevent auto-oxidation. 5 mL of aqueous leaf extract of the *S. bryopteris* was taken and added to 45 mL of prepared 1mM solution of AgNO_3_ and kept on stirrer at RT. After 24 h, the AgNP suspension was centrifuged at 12,000 rpm for 15 min and washed with distilled water thrice to remove any undesired plant extract.

### Optimization parameters for AgNP synthesis

The reaction mixture was monitored using different concentration of AgNO_3_ (1, 2, 3, 5 and 7mM), pH (1, 2, 3, 5, 7 and 9), temperature [0, 4, 37 (RT), 55 and 75°C], volume of leaf extract (1, 3, 5, 7, 9 mL) and incubation time (0, 12, 24, 48 h and 7 days).

### Physicochemical characterization of green-synthesized AgNPs

#### UV spectroscopy

To characterize AgNP, an aliquot (2mL) was drawn from the reaction mixture and measured using Shimadzu UV–VIS spectrophotometer (UV-1900i; Japan) at a wavelength of 200–800 nm with 1 nm resolution.

#### Field emission scanning electron microscope (FESEM), energy-dispersive X-ray (EDX), and mapping analysis

To determine the morphological appearance of AgNPs, FESEM (Zeiss GeminiSEM 500, Germany) was used. 20 µL of AgNP suspension was spread onto clean glass slides and allowed to dry at RT. An inbuilt EDX detector was used to demonstrate the elemental compositions and distribution maps of AgNPs. The corresponding EDX spectra was also taken for the elemental analysis of AgNPs.

#### Dynamic light scattering (DLS) and zeta potential analysis

The hydrodynamic diameter and surface charge of AgNPs was analyzed by using Zeta sizer equipment (Malvern, UK). For this, AgNPs were diluted ten-fold using distilled water and then transferred into Zetasizer tubes at 25°C. The zeta potential of AgNPs was determined with water as dispersant. Zeta potential measurement was used to study the stability of AgNPs in aqueous suspensions. It helped to determine the surface potential and electrostatic stability of green-synthesized AgNPs.

#### Transmission electron microscopy (TEM)

To determine the shape and size of green-synthesized AgNPs, TEM (TALOS L120C, Thermo Fisher Scientific) was used. After centrifuging the solution of AgNPs, the pellet was washed three times with distilled water, diluted 10 times and sonicated for 15 min. Then, 10 μL of AgNP suspension was loaded onto standard carbon coated 300 mesh copper grid, allowed to dry at RT and subjected to TEM^31,33^.

#### Fourier-transform infrared spectroscopy (FTIR)

FTIR analysis was performed to analyze the functional group present in the leaf extract of *S. bryopteris* and their possible mechanism in the synthesis of AgNPs. The FTIR spectra was recorded in the transmission mode (4000–500 cm^–1^) by using a Thermo Scientific (Nicolet iS50) FTIR Tri-detector.

#### X-ray diffraction (XRD) analysis

The crystalline nature of AgNPs was determined through XRD (Rigaku Smart Lab SE, Tokyo, Japan, X-ray source Cu, 3KW) in the angle range of 10°–80°. The sample crystallinity was determined by comparing observed patterns with standard powder patterns defined by Joint Committee on Powder Diffraction Standards (JCPDS).

### pH and thermal stability of AgNPs

The pH stability of synthesized AgNPs was investigated by changing the pH of AgNPs to 3, 5, 9, 11 and 7 (stock AgNPs) with constant temperature at 37°C. The pH of the solution was maintained by using freshly prepared 10% HNO_3_ and 0.1 M NaOH^34–36^. The heat stability of AgNPs was checked at different temperatures such as 3°C, 55°C, 70°C and at RT 37°C (stock AgNP) at constant pH 7. Samples were thoroughly mixed and vortexed followed by UV–Vis Spectrophotometer analysis to observe the absorption spectra pattern of the reaction mixture after 24 h of incubation. The antifungal activities of these reaction mixtures were evaluated against *Candida* spp. by disc diffusion method and the diameter of zone of inhibition was evaluated in each case^22^. The experiment was done in triplicate.

## Antifungal and antibiofilm studies

### Fungal strains, media and growth conditions

A total of four reference strains of *Candida* species including *C. albicans* (ATCC 90,028), *C. krusei* (ATCC 6258), *C. parapsilosis* (ATCC 22,019), *C. glabrata* (ATCC 15,545) were used in this study. All *Candida* strains were stored as glycerol stock at -20°C and revived on Sabouraud’s Dextrose Agar (SDA) plate (1% peptone, 2% glucose, 2% agar). The cells were streaked on SDA plate and incubated at 37°C to get single colony. Yeast colonies of each *Candida* strain were subcultured on SDA plate, and incubated at 37°C for 24 h. After 24 h of incubation, 4–5 yeast colonies were transferred (with a sterile loop) to a test tube containing 3 mL of 0.9% saline solution. The resulting fungal suspensions were vortexed for 15 s. The fungal cells were adjusted to the desired density for antibiofilm assay (1 × 10^7^ cells per mL at 0.1 optical density (OD) at 600 nm).

### Drug susceptibility testing

#### Determination of minimum inhibitory concentration (MIC)

Antifungal potency of green-synthesized AgNPs was determined by broth microdilution method in accordance with the guidelines recommended by the Clinical and Laboratory Standard Institute, following M27-A3 protocol^37^. Initially, ten concentrations (twofold) for the drugs were prepared in YPD broth media (1% yeast extract, 2% peptone, 2% dextrose) and a volume of 100 µL of each dilution was added to 96-well plate (Tarsons). Inoculum of all *Candida* strains was prepared as described above. Overnight grown cells, resuspended in normal saline (0.9%) to achieve an optical density (OD) of 0.1 at 600 nm, which corresponds to 1.0 × 10^7^ cells/mL, were used. These cells were further diluted in YPD media to attain a final concentration of 1.0 × 10^4^ cells/mL and a volume of 100 µL of cell suspension was added to each well of 96 well microtiter plate. Simultaneously, effect of AgNO_3_, leaf extract, FLU and AmB on *Candida* cells were also checked (controls). The plates were incubated at 37°C for 24 h. After incubation, the presence or absence of growth was observed visually. MIC was defined as the lowest concentration that produced visible (50%) inhibition of yeast growth. MFC was defined as the lowest concentration of the test compound that completely inhibited (100%) the growth of yeast. MIC, MFC values were duly recorded in each case.

#### Growth studies

Primary culture was prepared by inoculating 1 × 10^7^ *Candida* cells (OD_600_ 0.1) into 25 mL YPD broth, along with MIC concentration (0.003 ng/mL) of the green-synthesized AgNPs. Cells were grown at 37°C, with constant shaking at 120 rpm and growth was monitored at a regular interval of 2 h up to 24 h. Growth was measured by taking OD at 600 nm using Labomed Inc. Spectrophotometer.

#### Microdilution checkerboard assay

The antifungal activity of AgNPs in combination with a standard antifungal drug was evaluated by the checkerboard microdilution assay. 50 µL of AgNP (0.0486 to 0.00009 ng/mL) and 50 µL of fluconazole (FLU) (64 to 0.125 µg/mL) were added to 96 well microtitre plate. Each well was inoculated with 100 µL of intrinsically FLU resistant *C. krusei* (1 × 10^4^ cells/mL) to make up a final volume of 200 µL. The obtained checkerboard plates were incubated overnight at 37°C. The Fractional Inhibitory Concentration (FIC) of each compound was determined by the ratio of the MIC obtained when the compounds were tested in combination vis a vis the MIC of compounds when tested individually. The fractional inhibitory concentration index (FICI) was calculated using the following equation^38^:

$$\text{FIC Index}=\frac{\text{MIC of AgNP in combination}}{\text{MIC of AgNP alone}}+\frac{\text{MIC of Fluconazole in combination}}{\text{MIC of Fluconazole alone}}$$

where synergy and antagonism were defined by FICI ≤ 0.5 and > 4 respectively. Partial synergistic was defined by 0.5 > FICI < 1, whereas indifferent was defined by 1 < FICI ≤ 4.

#### Scanning electron microscopic (SEM) analysis to check cell surface morphology

To determine morphological changes in fungal cells in the presence of green-synthesized AgNPs, SEM (JEOL Japan Mode: JSM 6610LV) was used. Samples were prepared as described previously^39^. A single colony of selected strains grown on SDA was inoculated on YPD broth and incubated for 17 h at 37^◦^C at 200 rpm. Overnight culture was transferred into a fresh YPD broth to get an OD_600_ = 0.1. One half volume of this inoculum was exposed to MIC concentration of AgNPs (0.003 ng/mL) for 24 h and other half was used as control. Following the incubation, the cells were centrifuged, and washed with phosphate buffer saline (PBS) three times to remove unwanted residues at 5000 × g for 5 min. The obtained fungal pellet was fixed with 500 μL of 2.5% (w/v) of glutaraldehyde for 2 h at RT and washed twice with PBS. Thereafter, the cell-pellets were dehydrated in a graded ethanol series (30%, 50%, 70%, 90%) for 10 min in each grade and final dehydration was done with 100% ethanol. The final dehydrated cells were then added to circular glass coverslips for imaging in SEM. The fungal cells, grown in the absence of AgNPs were run with the same protocol and taken as negative control. Finally, changes in the fungal cell morphology were observed using SEM and the images were recorded**.**

#### Leakage of nuclear material

The impact of green-synthesized AgNPs on intracellular leakage of nuclear content from fungal cell was studied. Approximately 1 × 10^7^ *Candida* cells (OD_600_ 0.1) were resuspended in 5 mL of PBS. AgNPs were added at MIC and 2MIC values (0.003 and 0.006 ng/mL respectively) to the *Candida* cells and incubated at 37°C at 120 rpm for 12 h. After 24 h, aliquots of 1.5 mL were taken out and centrifuged for 5 min at 10,000 rpm. Finally, 1 mL of supernatant was taken and absorbance was recorded at 260 nm using Shimadzu UV–VIS spectrophotometer (UV-1900i)^40^. The untreated *Candida* cells were prepared as above and used as control, along with the treated cells.

#### Mitochondrial activity

To check the effect of green-synthesized AgNPs on mitochondrial activity, MTT (3- (4,5-cimethylthiazol-2-yl)-2,5-diphenyl tetrazolium bromide) assay was performed as described previously^41^. Overnight culture of *Candida* cells was diluted in fresh YPD medium to get an initial OD_600_ of 0.1. The culture was then treated with MIC and 2MIC concentrations (0.003 and 0.006 ng/mL respectively) of AgNPs for 24 h and controls (untreated cells) were prepared in the same manner. Following two PBS washes, 500 µL of suspensions was collected, to which was added 500 µL of MTT (100 µg/mL) and incubated for 4 h. Cells were again harvested and washed twice with PBS. After that, pellets were suspended in 1 mL of DMSO (dimethyl sulfoxide) and shaken for 5 min at 37°C. The suspensions were centrifuged, and OD_570_ of the supernatants was measured by using Shimadzu UV–VIS spectrophotometer (UV-1900i).

#### Ergosterol quantification

A single *Candida* colony from overnight SDA plate was inoculated onto YPD media and incubated at 37°C for 24 h under constant agitation at 200 rpm. The overnight cultures were transferred to fresh YPD broth to get OD_600_ = 0.1. The cultures were further transferred to four different flasks and tested against MIC AgNPs (0.003 ng/mL), 2MIC AgNP (0.006 ng/mL), FLU (positive control) along with untreated cells (negative control). The contents were incubated at 37°C for 24 h with constant shaking at 200 rpm. The cells were then harvested by centrifugation at 2700 rpm for 5 min and washed thrice with sterile distilled water. The net weight of cell pellet was determined. Each pellet was mixed with 3 mL of an alcoholic potassium hydroxide solution (25g of KOH; and 35 mL of distilled water, brought to 100 mL with absolute ethanol), and vortexed for 1 min to extract the sterols. The cell suspensions were transferred to sterile borosilicate glass screw-cap tubes and incubated at 85°C for 1.5 h. Following incubation, the glass tubes were allowed to cool at RT. Sterols were extracted by addition of a mixture of 1 mL of sterile distilled water and 3 mL of n-heptane (Thermo Fisher Scientific) followed by vigorous mixing of the solution for 3 min. Heptane layer (upper transparent layer) containing ergosterol and 24(28)-dihydroxy-ergosterol (DHE) was scanned spectrophotometrically between 240 and 300 nm using Shimadzu UV–VIS spectrophotometer (UV-1900i). Both ergosterol and 24(28)-DHE absorbs at wavelength 281.5 nm, whereas only 24 (28) DHE absorbs at wavelength 240 nm. Ergosterol content is determined by subtracting the amount of 24(28)-DHE (calculated from the OD_240_) from the total ergosterol plus 24(28)-DHE content (calculated from the OD_281.5_)^42^. Ergosterol content was calculated as percentage of the wet weight of the cells using the following equations:

% ergosterol + % 24(28)-DHE = [(A281.5/290) × *F*]/pellet weight.

% 24(28)-DHE = [(A240/518) × *F*]/pellet weight and late intermediate.

% ergosterol = [% ergosterol + % 24(28) DHE] − % 24(28) DHE, where *F* is the factor for dilution in petroleum ether and 290 and 518 are the *E* values (in percent per centimeter) determined for crystalline ergosterol and 24(28)-DHE, respectively.

#### Biofilm analysis

##### Biofilm total biomass quantification-crystal violet (CV) staining assay

To check the antibiofilm potency of the green-synthesized AgNPs, biofilm inhibition assay was performed as described previously^43^. *Candida* biofilms were produced on pre-sterilized, polystyrene, flat-bottom 24-well microtiter plate. A loopful of yeast cells from the SDA plate were inoculated into flasks containing YPD liquid media (usually 20 mL of media in a 150 mL flask) and incubated overnight in an orbital shaker (150 rpm) at 37°C. Cells were harvested from overnight-grown liquid cultures by centrifugation (approximately 3,000 rpm for 5 min at 4°C), supernatant was removed and pellet was washed twice with sterile PBS. The cell cultures were adjusted to an optical density of 0.1 (OD_600_ nm). From, the prepared inoculum, 1 mL of fungal suspension was dispensed into the selected wells of the 24-well microtiter plate. When all the selected wells have been seeded, microtiter plate was covered with its original lid, sealed with parafilm and placed inside an incubator at 37°C for 24 h. After 24 h, biofilms exhibited the complex three-dimensional structural architecture of multicellular communities and can be used for further antifungal susceptibility testing. Biofilm were washed thrice with sterile PBS carefully without disrupting its structure, to remove planktonic or non-adherent cells that remain in the wells. *Candida* biofilms were treated with MIC, 2MIC conc. of AgNPs (0.003 and 0.006 ng/mL respectively) along with control (biofilm not exposed to AgNPs). After this, the microtiter plates were incubated for 24 h at 37°C. Subsequently, the effect of AgNPs on the formation of *Candida* biofilm was assessed by CV staining assay. The CV assay involves crystal violet which is a basic dye that binds to extracellular matrix (ECM) of the biofilm. The dye works on the principle that biofilm cells that undergo cell death will lose their adherence and are lost from the population of cells, reducing the amount of CV staining. The dye retained by the biofilm (in the destaining solution) may be measured spectrophotometrically.

For this, wells of microtiter plates were washed three times with 200 μL of PBS and air dried for 45 min. The biofilm biomass attached to the wells was determined by staining with 100 μL of 0.4% (w/v) CV solution for 45 min. Excess CV solution was removed by washing carefully with 200 μL of sterile PBS (three time) and then destained with 200 μL of ethanol. After 45 min of destaining, 100 μL of the destaining solution was transferred into the wells of new microtiter plate and the amount of CV stain in the destaining solution was measured at OD_595_ nm. The absorbance values for the controls were subtracted from the values for the test wells to minimize background interference. The percentage of biofilm reduction was calculated by following formula:

Biofilm Inhibition (%) = [(Control_OD_–Treated_OD_)/Control_OD_] × 100.

##### Biofilm structure visualisation

In order to visualize the structure of biofilm in the presence (MIC and 2MIC) and absence of AgNPs, SEM was performed. For this, biofilm was formed on circular glass coverslips as described above and were fixed with 4% of glutaraldehyde for 1 h at RT. After this, biofilm was dehydrated with graded series of ethanol (30, 50, 70, 90%) for 15 min in each alcohol graded solution. The final dehydration was done using 100% ethanol for 15 min and then air dried for 20 min. Prior to observation, the coverslips containing biofilm were mounted on stubs, sputter coated with gold by using EMITECH K550X sputter coater and then observed with ZEISS EVO 18 SEM^44,45^.

### Statistical analysis

ANOVA (Fischer’s and Welch’s) was conducted along with post-hoc (Tukey’s and Games-Howell’s analysis). Levene’s test was conducted to check for heteroskedasticity in data. The analyses were conducted using Jamovi statistical software. ImageJ software (National Institutes of Health, Bethesda, MD, USA) was used to measure AgNPs particle diameter, and Origin 8 (Origin Lab Corporation, Northampton, MA, USA) was used to obtain all physicochemical analysis data.

## Results

### Synthesis and characterization of green-synthesized AgNPs

**Fig. 1:**
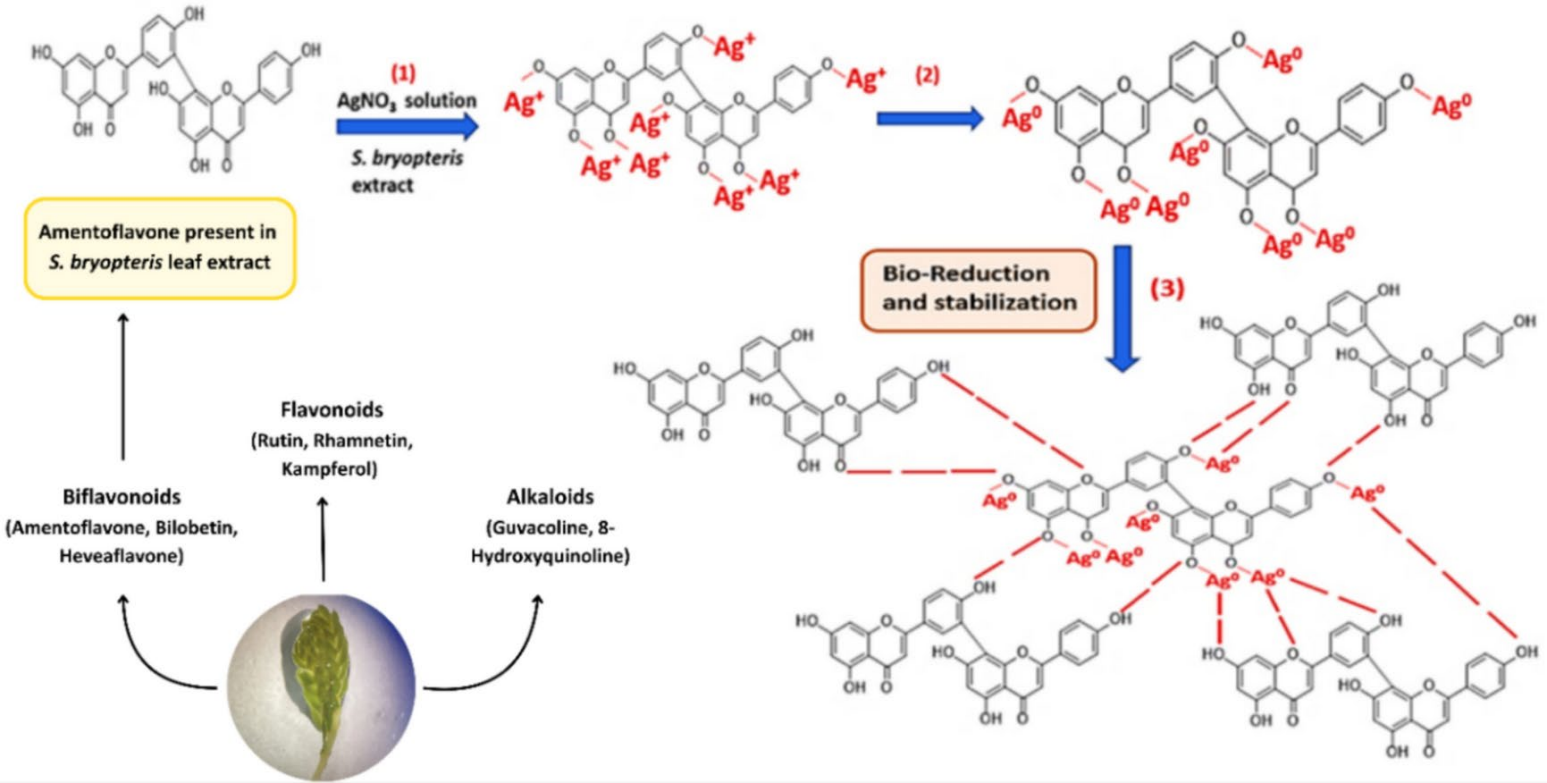
Possible mechanism of the action of the reducing agent(s) of *S. bryopteris* leaf extract (containing amentoflavone) causing Ag^+^ ions to form green-synthesized AgNPs.

Previous research on *S. bryopteris* has led to the identification of various phytochemicals present in the leaf extract including amentoflavone, heveaflavone, rutin, syringresinol, bilobetin, hinokiflavone, 2,3-dihydroamentoflavone, 2,3-dihydrohinokiflavone, tetrahydroamentaflavone, tetrahinokiflavone, lanaroflavone, sciadopitysin, sequoiaflavone, tetrahydrohinokiflavone, imidazole, palmitic acid, L-fucitol, gallic acid, lupeol, catechine, myo-inositol^46,47^. These phytochemicals play important role in the reduction, capping and stabilization of AgNPs (Fig. 1).

**Fig. 2:**
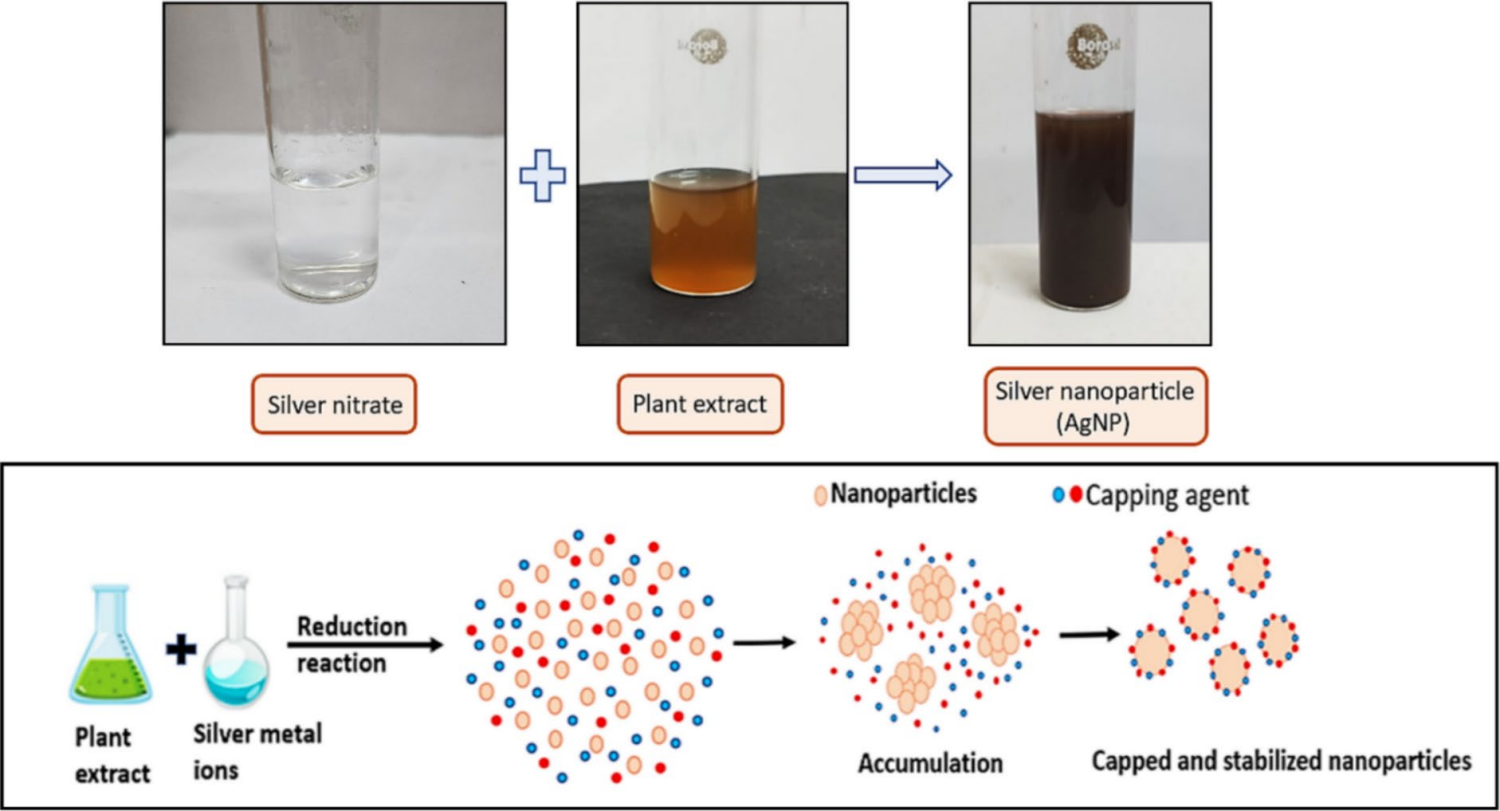
Synthesis of AgNPs using leaf extract of S. *bryopteris* and AgNO_3_ (1mM).

The green synthesis of AgNPs was confirmed by the colour change from pale yellow to deep red brown (Fig. 2). The colour change indicates the visible sign of the initiation of reaction followed by nucleation and growth of silver NPs where neighbouring nucleonic particle integrate with each other to make thermodynamically stable AgNPs.

**Fig. 3:**
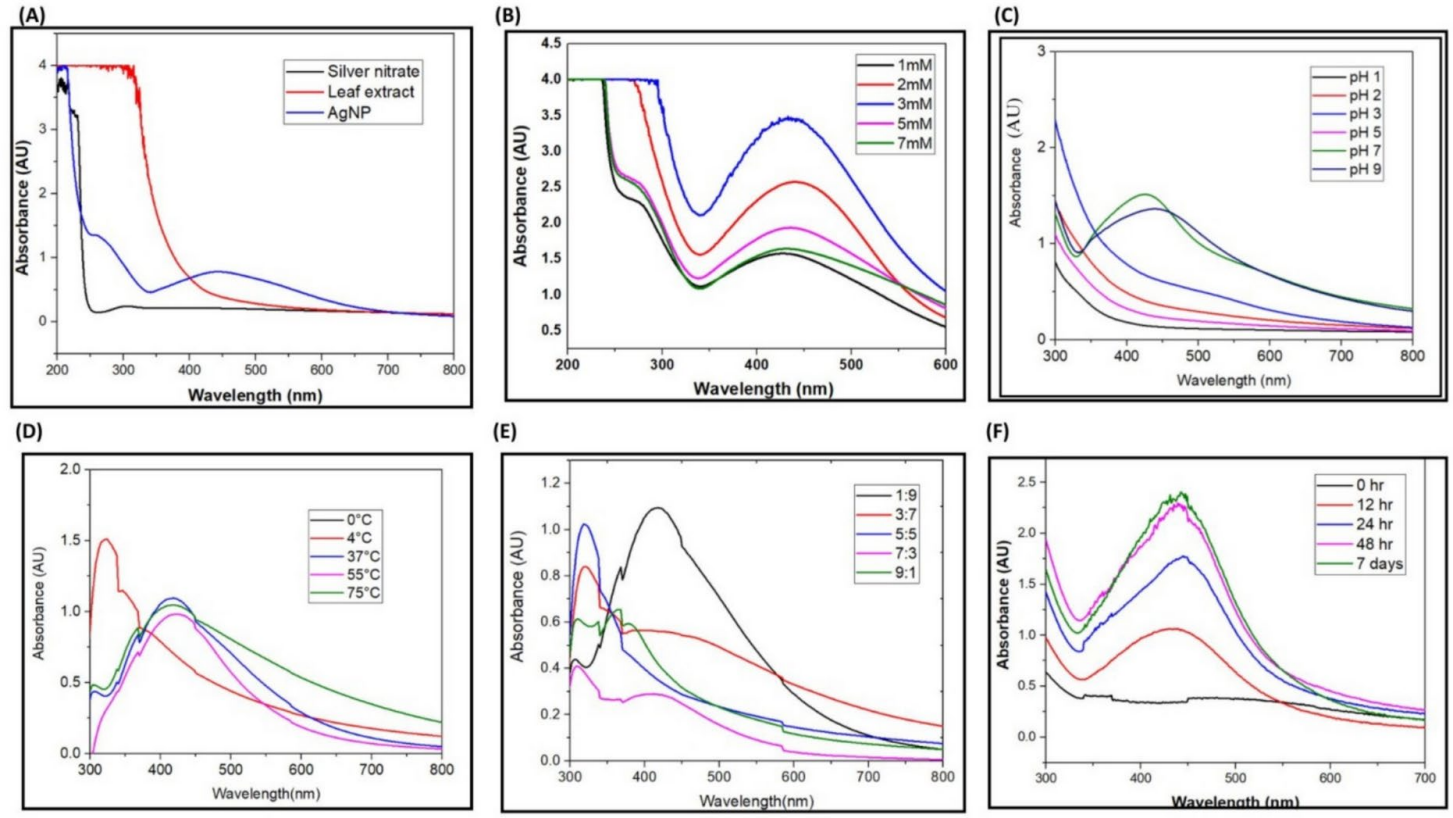
UV–Visible spectroscopy showing absorption spectrum of (**A**) leaf extract, 1mM silver nitrate, AgNPs; and Effect of (**B**) silver nitrate concentration (**C**) pH (**D**) temperature (**E**) ratio of plant extract to AgNO_3_ (**F**) incubation time on the synthesis of AgNPs.

The synthesis of AgNPs was completed in two steps, firstly, Ag^+^ ions from the AgNO_3_ is reduced to Ag^0^ (elemental form), by the secondary metabolites present in the plant extract that acts as biological catalysts. The oligomeric clusters are formed by Ag^0^ through agglomeration that led to formation of AgNPs (Fig. 2). The broad peak obtained in the visible range is due to the change in colour occurring because of the excitation of free electrons responsible for the establishment of the Surface Plasmon Resonance (SPR) band as the conduction and valence band of metallic NPs lie close to each other. The SPR band can be used to determine the colour, size, shape and morphology of AgNPs^48^. The characteristic SPR band obtained at 425 nm suggests the complete reduction of elemental silver to AgNPs (Fig. 3A). This finding is in agreement with the previous studies confirming the formation of AgNPs at around 400–450 nm^49,50^.

Several factors were optimized for the synthesis of AgNPs including concentration of AgNO_3_, volume of plant extract, temperature, pH and reaction time that lead to fabrication of NPs in different sizes and shapes and can further affect antifungal activity of AgNPs. The increase in concentration of AgNO_3_ from 1 to 3 mM led to faster reduction of Ag^+^ to Ag^0^, but the increase in concentration from 5 to 7 mM has resulted in the reduction of peak intensity which can be explained by the formation of agglomerated AgNPs. It has been studied that width of each SPR in UV–Visible spectra is related to the size distribution of colloidal AgNPs^51^ (Fig. 3B). SPR plays a major role in the determination of optical absorption spectra of metal NPs, which generally shifts to a longer wavelength (red shift) with increase in particle size of NPs. With the increase in concentration of AgNO_3_, the peak sharpness was reduced and shifted towards right side indicating redshift. Widening of peak or red shift shows the aggregation of NPs resulting in their bigger size. At low concentration of AgNO_3_, plant extract completely reduces them into AgNPs, but at higher concentrations, it does not get completely reduced and leads to the formation of large sized NPs due to the aggregation of unreacted AgNO_3_ with AgNPs^52^. Hence, 1 mM concentration of AgNO_3_ is used for further studies as at this concentration of AgNO_3_ the size of AgNPs was most favourable.

The pH of reaction solution plays a crucial role in the synthesis of metal NPs. The increase in pH of reaction mixture increases the nucleation, and agglomeration of NPs and the rate of reaction by reacting with the functional group of secondary metabolites that plays major role in capping and stabilization of NPs (Fig. 3C). At low pH there was no indication for the synthesis of AgNPs in UV–Vis spectra, the presence of higher positive charges at low pH on the surface of NPs help to attract negative charge based phytocompounds and leads to flocculation, while in the case of alkaline pH, the presence of hydroxyl ions on the surface of NPs exerts dominant repulsive force to colloidal solution and particle size or agglomeration gets reduced. Thus, pH 7 was found to be the optimal pH for the synthesis of NPs.

The reaction temperature also had significant effects on the synthesis, morphology of AgNPs. In this study, we have evaluated the green synthesis of AgNPs at different temperatures including 0, 4, 37 (at RT), 55 and 75°C. (Fig. 3D). At 0 and 4°C there was no significant peak observed in the absorption spectra indicating that there is no formation of AgNPs. At RT, 55 and 75°C the absorption peak was observed, indicating the formation of AgNPs. It has been suggested that at high temperature, the kinetic energy of molecules increases and silver ions reacts rapidly with phytocompounds of leaf extract and leads to the formation of smaller sized NPs^53^. 37°C (RT) is identified as optimal temperature for the synthesis of optimal-sized AgNPs.

The ratio of leaf extract to AgNO_3_ (1 mM) was optimized as 1:9, 3:7, 5:5, 7:3, 9:1 (Fig. 3E). On increasing the volume of leaf extract into AgNO_3_ solution, the colour of the reaction mixture become blackish grey and the formation of AgNPs was prevented. From the UV–Vis absorption spectra of AgNPs it has been clearly demonstrated that 1:9 ratio of leaf extract and silver nitrate exhibited optimal and best synthesis of AgNPs and absorption peak was observed at 425 nm.

In this study, the formation of AgNPs was examined by measuring the absorption spectra at regular time intervals. At the start of the reaction, ie 0 h, there was no formation of AgNPs. From 12 h, the peak at 425 nm began to form and this showed the formation of AgNPs. After this, increase in absorption bands was observed due to the enhanced synthesis of AgNPs. An increase in absorbance was observed with the passage of time showing enhancement of synthesis of AgNPs. So, the best optimum condition for the completion of reaction was considered as 12–48 h (Fig. 3F).

Thus, it is clearly evident from the results, the optimal parameters required for the synthesis of AgNPs from leaf extract of *S. bryopteris*, is 1mM concentration of AgNO_3_, 1:9 ratio of leaf extract: AgNO_3_, at 37°C (RT) at neutral pH, for 48 h.

### FESEM and EDX analysis

**Fig. 4:**
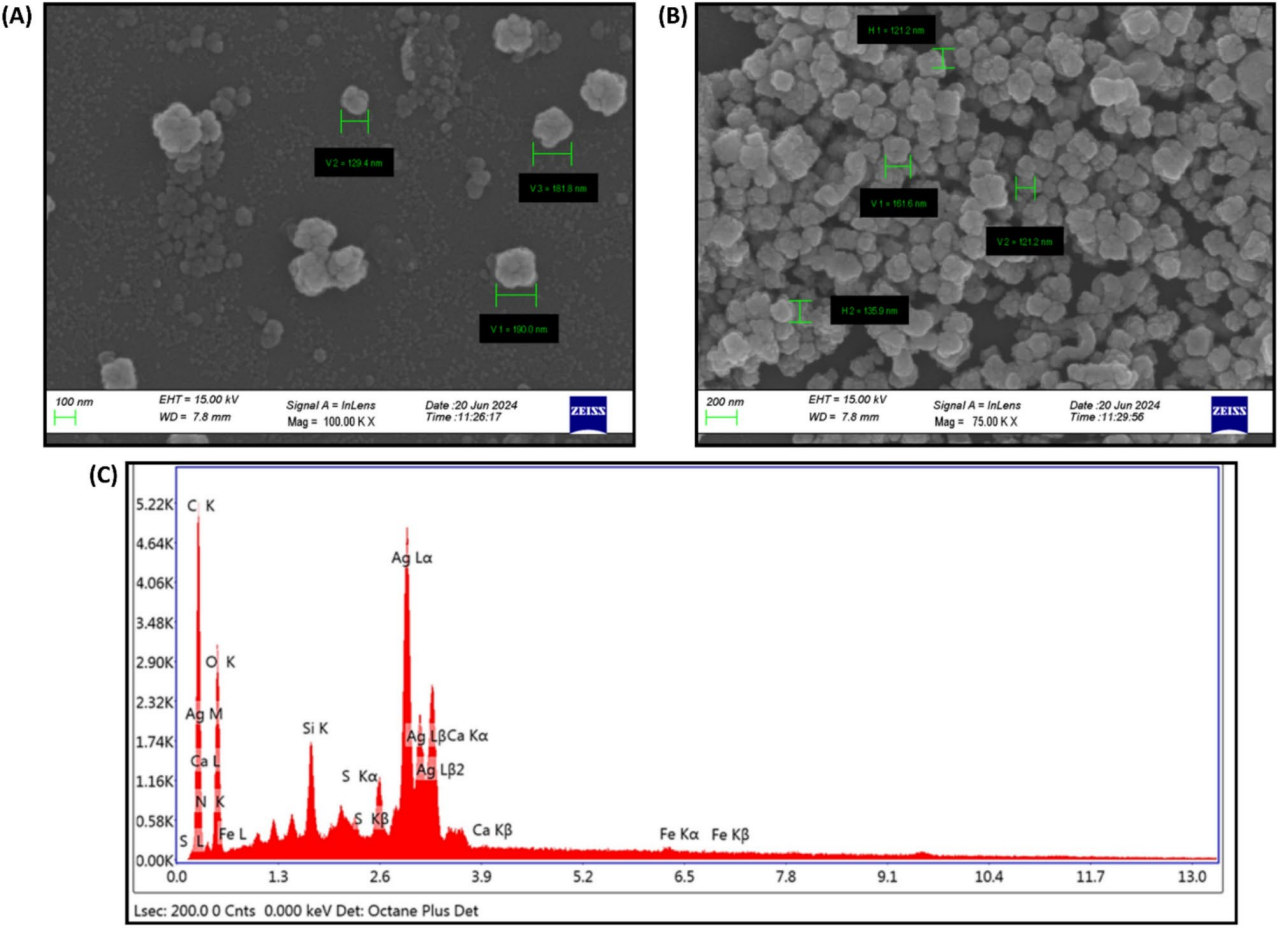
(**A**) and (**B**) FESEM images of AgNPs synthesized from leaf extract of *S. bryopteris* at different magnifications (100 and 75 KX); (**C**) EDX analysis of *S. bryopteris* derived AgNPs showing peak of silver at 3 keV.

FESEM micrographs have shown that green-synthesized AgNPs were oval and spherical in shape (Fig. 4A and B).

Additionally, EDX was used to determine the elemental composition and purity of AgNPs. The primary constituent of AgNP is silver (Ag), carbon (C) and oxygen (O) (Table 1). The absorption bands corresponded to that of C and O elements that are characteristic of *S. bryopteris* leaf extract. These signals can be attributed to biomolecular corona surrounding AgNPs and phytochemicals from leaf extract that acted as capping and stabilizing agents during the synthesis of AgNPs^54^. Furthermore, EDX analysis demonstrated a sharp peak at 3keV, that corresponds to typical absorption of metallic or elemental silver (Fig. 4C).

**Table 1:** Elemental composition of *S. bryopteris* derived AgNPs.

Element	Weight %	Atomic %
C K	17.88	33.16
N K	0	0.01
O K	38.72	53.9
Si K	3.84	3.04
S K	2.43	1.69
AgL	35.32	7.29
CaK	1.23	0.68
FeK	0.57	0.23
Total	100	100

### DLS and zeta potential analysis

**Fig. 5:**
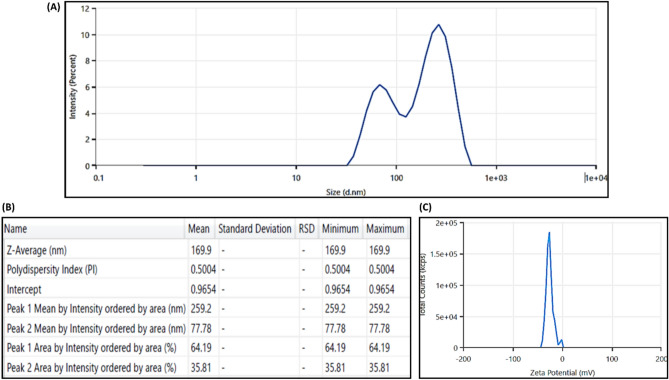
(**A**,**B**) DLS spectrum (**C**) Zeta potential of AgNPs synthesized using leaf extract of *S. bryopteris.*

The hydrodynamic diameter (HD) of green-synthesized AgNPs was determined by using DLS. The size of AgNPs plays an important role in determining its antifungal activity^55^. AgNPs with smaller size or with smaller dimensions are more capable to penetrate inside the cell wall of *Candida* and can cause immense destruction to the cells of fungal pathogens. Bigger size of NPs negatively affects their permeation through cellular membranes. HD of AgNPs was found to be 169.9 nm with a polydispersity index (PDI) of 0.5004. Green-synthesized AgNPs exhibited two HDs at 259.2 and 77.78 nm (Fig. 5A,B). However, the high HD values of AgNPs was attributed to the presence of biomolecules or secondary metabolites of *S. bryopteris* responsible for the reduction, capping and stabilization of AgNPs and water molecules of the aqueous system coating the AgNPs. DLS generally measures the HD of AgNPs in association with biological molecules (secondary metabolites of plant extract) and ions attached to the surface of AgNPs.

Zeta potential helped us to understand the surface charge present on NPs that corresponds to its stability. The zeta potential of green-synthesized AgNPs was found to be − 25.39 mV (Fig. 5C). The value demonstrated that the green-synthesized AgNPs have colloidal stability. The result of our study is concordant with previous studies, where the zeta potential of AgNPs synthesized from bacterial cellulose and cell free filtrate of *Komagataeibacter rhaeticus* N1MW322708 strain was found to be − 27.7 mV and -32.7 mV^56^. The high value of zeta potential indicates a higher electric charge on the surface of NPs, that can cause repulsive forces among NPs and can prevent agglomeration while lower zeta potential values encourage aggregation because of Vander Waals interactions between NPs, resulting in larger size with lower antifungal activity. The major factor that determines the stability of AgNPs are the charge and secondary metabolites present on their surface that help in coating and capping of AgNPs. AgNPs is considered as stable nanosuspension when their zeta potential value is around ± 30 mV.

### TEM analysis

**Fig. 6:**
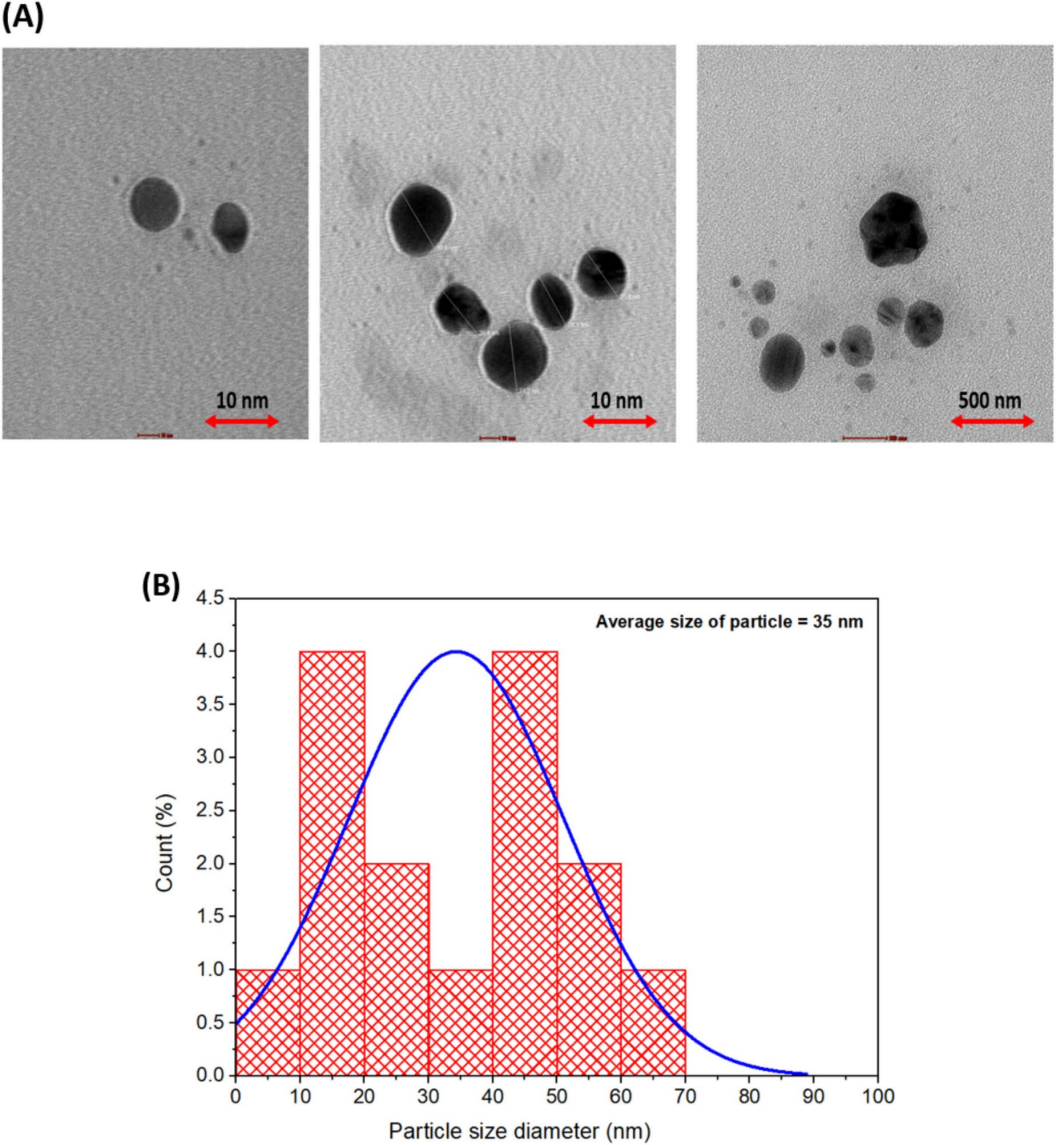
(**A**) TEM micrograph and (**B**) Particle size distribution/histogram of green-synthesized AgNPs.

TEM micrographs showed that AgNPs have a uniform spherical shape (Fig. 6A). The average diameter of green-synthesized AgNPs was found to be 35 nm in size. The TEM micrograph showed that NPs were well dispersed or monodispersed particles, without any agglomeration. The size of synthesized AgNPs was measured with the help of ImageJ software (Fig. 6B). The differences in the size of AgNPs between DLS and TEM findings is because DLS measures the size of NPs in aqueous medium while TEM measures it in a dry state. DLS is very sensitive in nature as it measures every motion of AgNPs in aqueous sample. Brownian movement affects the size of NPs as these particles are present in hydrated form and contributes to increased light scattering that lead to larger values of NPs. In addition to this, the hydrodynamic diameter is also affected by the secondary metabolites present in plant extract which are required for the coating, reduction and stabilization of NPs.

### FTIR analysis

**Fig. 7:**
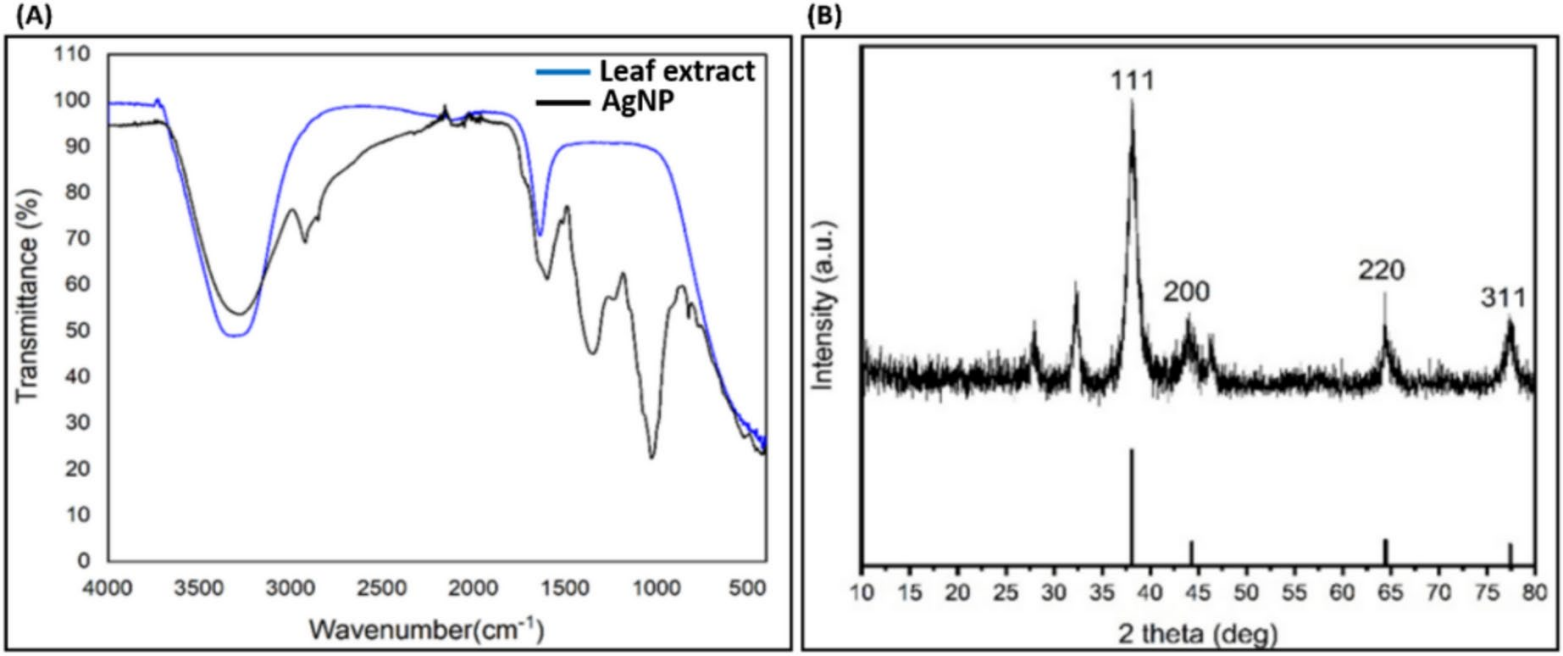
(**A**) FTIR and (**B**) XRD pattern of green synthesized AgNPs.

FTIR analysis was performed to determine the functional group of capping and stabilizing agents i.e. phytocompounds on the surface of NPs (Fig. 7A). AgNPs showed distinct spectral peak at 3250 cm^-1^ showing the symmetric vibration of O–H, while the peak observed at 2928 cm^-1^ showed asymmetric vibration of aliphatic C-H bonds. The peaks observed at 1628 cm^-1^ indicates the presence of C=O bond and peak at 1475 cm^-1^ is categorised as C=C, peak obtained at 1352 cm^-1^ is related to C-N bonds. In addition to this, peaks obtained at 1180 cm^-1^ indicates the presence of O–H groups of phenols^50,57^. The characteristic functional groups obtained in the result indicate that flavonoids present in the leaf extract of *S. bryopteris* have participated in the bio-reduction of Ag^+^ into AgNPs.

### XRD analysis

The 2Ɵ and corresponding miller indices (hkl) (Fig. 7B) are indexed to the face centred cubic (FCC) structure of AgNPs (metallic silver) (JCPDS file no 04-0783). The peaks obtained were matched from the Joint Committee on Powder Diffraction Standards (JCPDS) database. The XRD analysis showed characteristics peaks at around 38.1, 44.3, 64.4, 77.4 for AgNPs which were attributed to (111), (200), (220) and (311) corresponding planes. The obtained peaks indicate the highly crystalline nature of AgNPs. The additional diffraction peaks observed can be linked to organic phytocompounds present on the surface of AgNPs^58^

### *In-vitro* studies

#### Evaluation of antifungal activity of AgNPs

The MIC and MFC values of green-synthesized AgNPs against *Candida* spp. were found to be 0.003 and 0.006 ng/mL respectively (Table 2). MIC test was performed to determine the minimum inhibitory concentration of green-synthesized AgNPs against *Candida* spp. at ten different concentrations. In our study, we have reported that green-synthesized AgNPs work as potent antifungal agent against all *Candida* spp. at very low concentration in comparison to commercially available antifungal drugs.

**Table 2:** In vitro antifungal susceptibility testing of green synthesized AgNPs.

Name of fungal strains	AgNPs (ng/mL)		AgNO_3_ (µg/mL)		Leaf extract (µg/mL)		FLU (µg/mL)		AmB (µg/mL)	
	MIC	MFC	MIC	MFC	MIC	MFC	MIC	MFC	MIC	MFC
*C. albicans* (ATCC 90,028)	0.003	0.006	10.61	21.2	250	500	2	4	1	2
*C. krusei* (ATCC 6258)	0.003	0.006	10.61	21.2	250	500	16	32	1	2
*C. glabrata* (ATCC 15,545)	0.003	0.006	10.61	21.2	250	500	1	2	0.5	2
*C. parapsilosis* (ATCC 22,019)	0.003	0.006	10.61	21.2	250	500	1	2	0.5	2

Untreated control cells showed normal growth when compared to AgNPs treated cells.

**Fig. 8:**
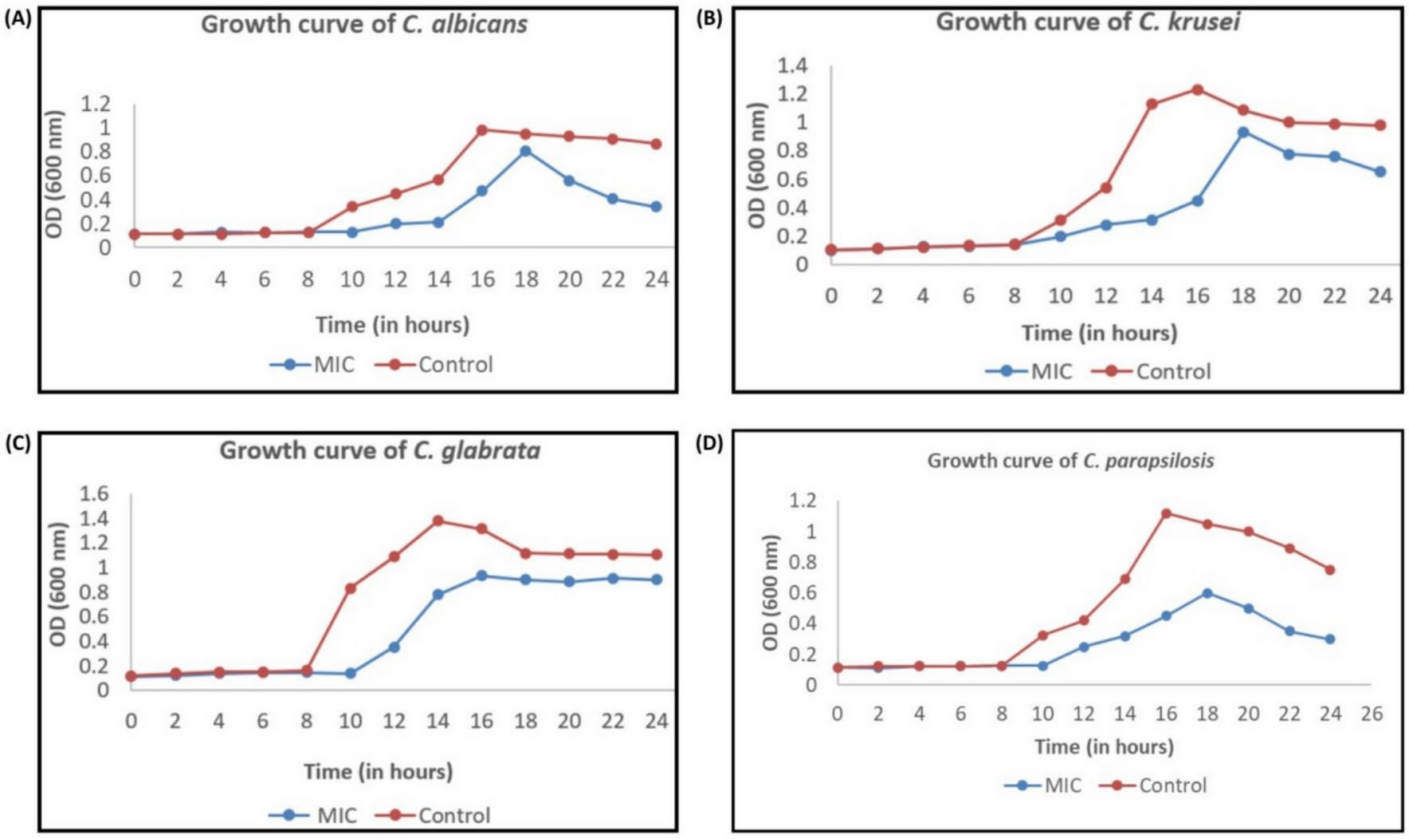
Growth kinetics curve of (**A**) *C. albicans* (ATCC 90,028) (**B**) *C. krusei* (ATCC 6258) (**C**) *C. glabrata* (ATCC 15,545) (**D**) *C. parapsilosis* (ATCC 22,019) in the presence of MIC concentration of AgNP (0.003 ng/mL).

For the study of growth kinetics, cells of *C. albicans*, *C. glabrata*, *C. parapsilosis*, *C. krusei* were treated with green-synthesized AgNPs at its MIC value (0.003 ng/mL). In comparison to control, the growth of test organism was found to be very less in the presence of AgNPs. From the graph (Fig. 8), it is clearly evident that growth of *Candida* species rapidly reached exponential phase in the absence of green-synthesized AgNPs, while in the presence of AgNPs it took longer time to reach the same.

The synergistic mode of interaction is observed between the green synthesized AgNPs and commercially available antifungal drug FLU that can be attributed to the fact that both FLU and AgNPs have different cellular targets but enhances each other antifungal efficiency. The combinatorial therapy helps to reduce the commercial drug toxicity by decreasing the dose of drugs and also reduce the risk of emergence of drug-resistant fungal pathogens^54^. In this study, we have observed synergistic interaction between the green synthesized AgNPs and FLU, wherein the combined action of both antifungal drug and AgNPs is much higher than the action of single drug, indicating that they are targeting different cellular components^54,59–62^. The combined effect of AgNPs and FLU were evaluated by checkerboard method and FIC index values were calculated. The combinatorial dose of AgNPs with FLU has reduced more than fivefold times than MIC values of AgNP used alone and the mode of interaction between two was found to be synergistic (Table 3).

**Table 3:** FIC index values of AgNPs in combination with commercially available antifungal drug, FLU.

Name of strains	MIC					
	Alone AgNP (ng/mL)	Alone FLU (µg/mL)	Combination		FICI	Mode of interaction
			AgNP	FLU		
*C. krusei* (ATCC 6258)	0.003	16	0.0007	0.5	0.26	Synergistic

#### Stability and antifungal activity of AgNPs at different pH and temperature

**Fig. 9:**
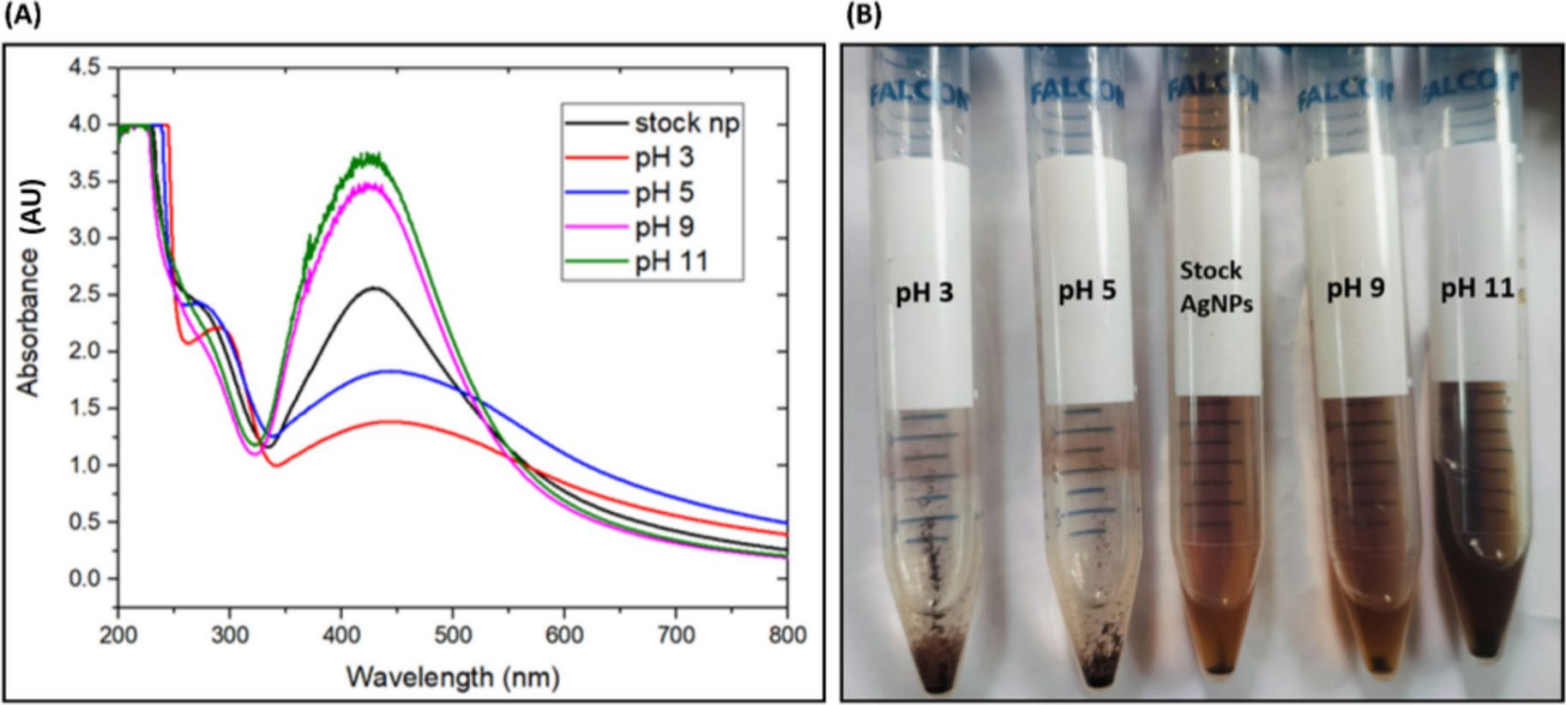
(**A**) UV–visible spectra of green-synthesized AgNP at different pH, (**B**) Visual appearance of AgNPs at different pH. (Stock AgNP is at pH = 7, temperature = 37°C).

The pH stability assay of AgNPs was carried out by subjecting the formed AgNPs to different pH (using 10% HNO_3_ and 0.1 M NaOH) solutions. Spectrophotometer assay was used to observe absorption spectra of AgNPs at pH 3, 5, 9, 11 after 24 h of incubation period. It is observed from Fig. 9A and B, the absorption peak was present at 425 nm in all pH conditions although pH 9 and 11 showed higher absorbance as compared to pH 3 and 5.

**Fig. 10:**
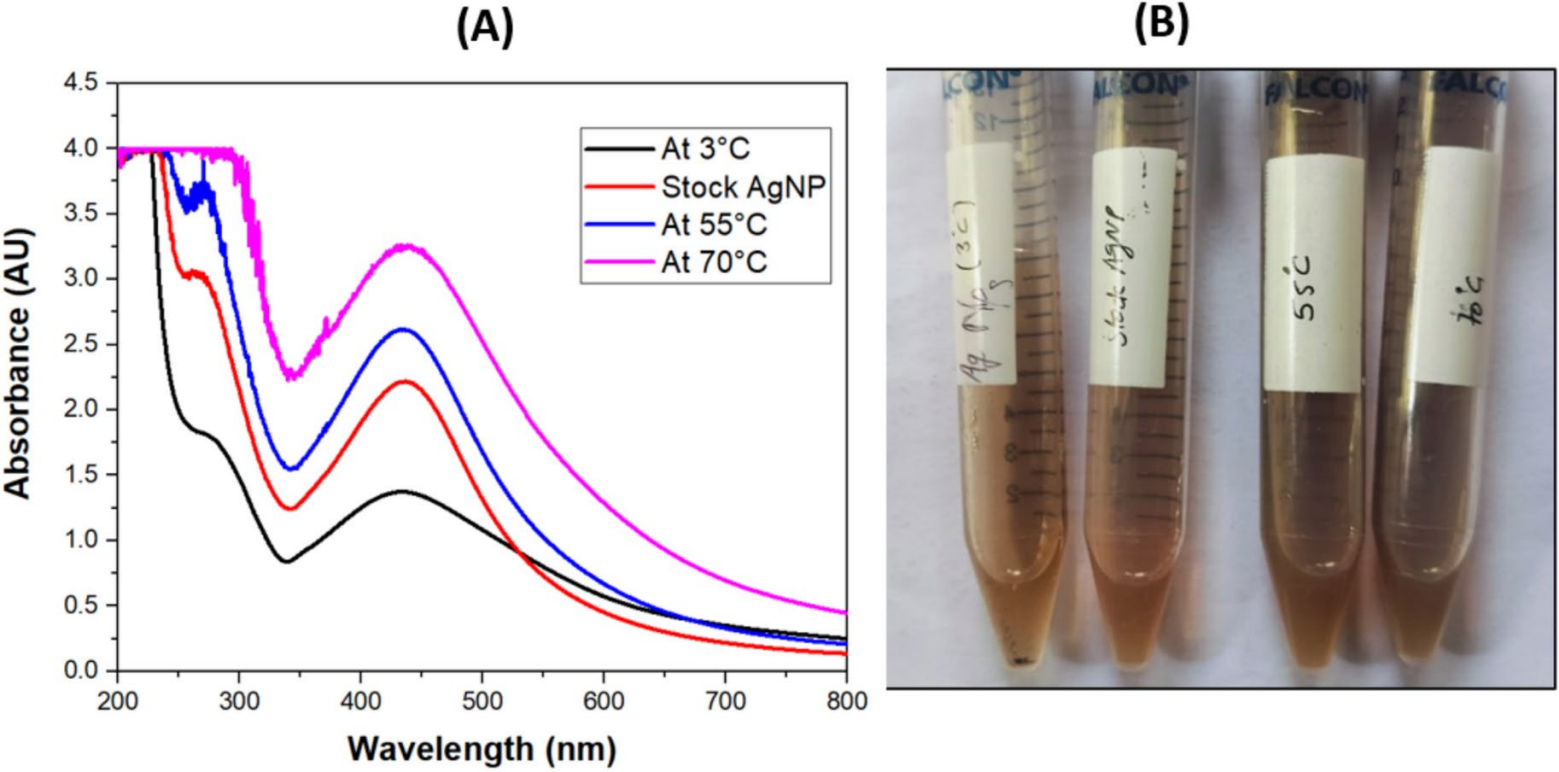
(**A**) UV–visible spectra (**B**) Visual appearance of AgNPs at different temperature to investigate stability of green-synthesized AgNPs. (Stock AgNP is at pH = 7, temperature = 37°C).

The heat stability of synthesized AgNP was checked by subjecting the AgNPs to different temperatures i.e. 55°C and 70°C and at 3°C for an incubation period of 2 h and their absorption spectra were recorded. From Fig. 10A and B, it is observed that the absorption peak was present at 425 nm in all temperature conditions though the magnitude of the peak increased with increase in temperature. These results indicated the thermo-tolerant nature of the AgNPs. Further their stability was confirmed by the retention of the antifungal activity of these AgNPs post their exposure to different pH and temperatures.

**Fig. 11:**
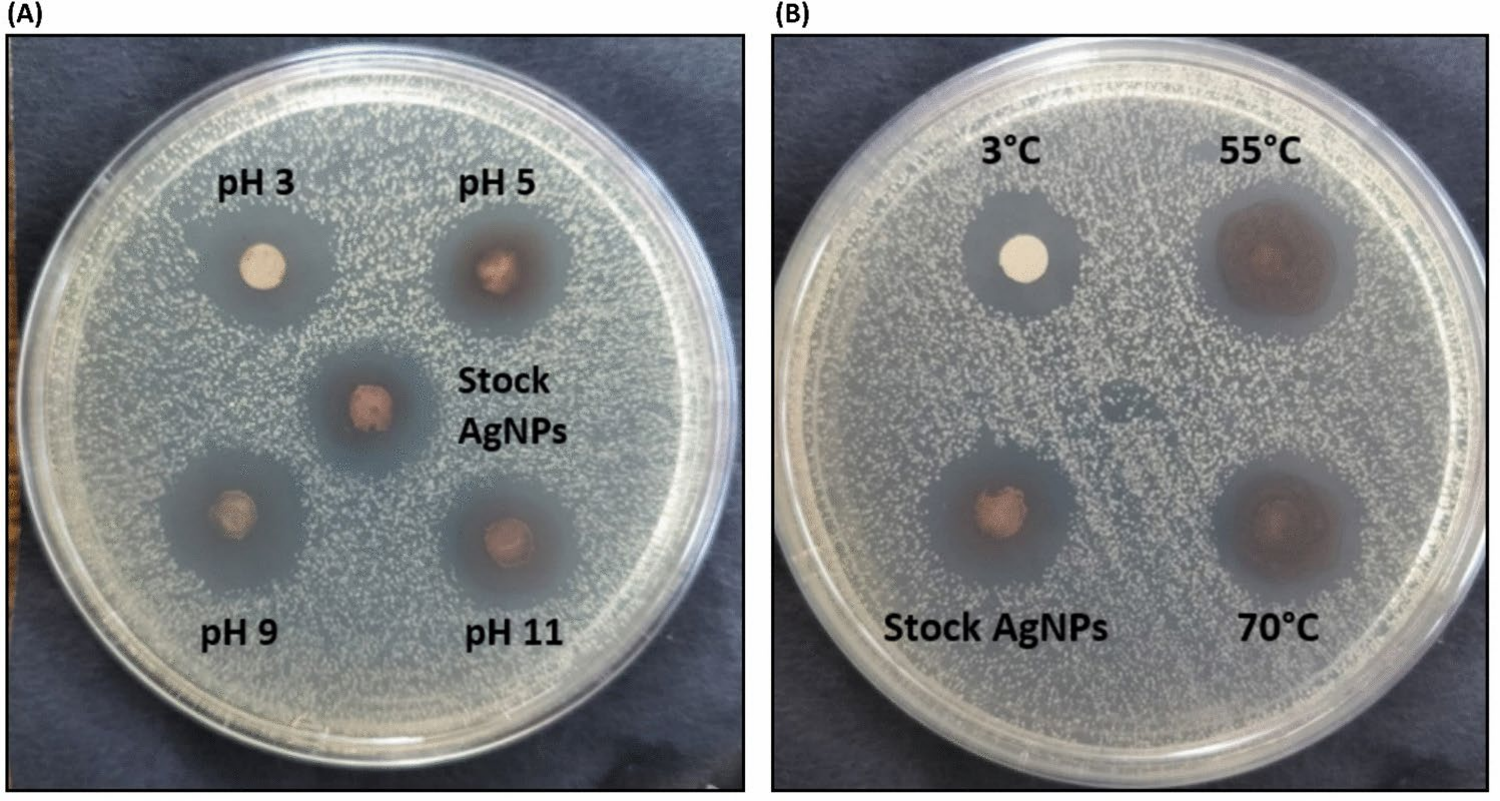
Antifungal activity of AgNPs against *C. glabrata* (ATCC 15,545) at different (**A**) pH (**B**) temperatures (Stock AgNP is at pH = 7, temperature = 37°C).

The antifungal activity of AgNPs exposed to different pH (with constant temperature) and at different temperature (with constant pH), was evaluated against *C. glabrata* by agar disc diffusion method (Fig. 11**, **Table 4). The zone of inhibition (ZOI) was measured and expressed as mean ± SEM.

**Table 4:** Diameter of zone of inhibition (in cm) of *Candida* spp at different pH and temperature.

Tested strain	Treatments given to AgNPs							
	pH 3/37°C	pH 5/37°C	pH 9/37°C	pH 11/37°C	Stock AgNPs (pH 7, temperature 37°C)	3°C/pH 7	55°C/pH 7	70°C/pH 7
*C. albicans* (ATCC 90,028)	1.5 ± 0.01	1.58 ± 0.005	1.68 ± 0.005	1.89 ± 0.005	1.51 ± 0.01	1.1 ± 0.05	1.91 ± o.oo5	1.7 ± 0.006
*C. krusei* (ATCC 6258)	1.48 ± 0.005	1.58 ± 0.01	1.68 ± 0.005	1.89 ± 0.01	1.48 ± 0.005	1.1 ± 0.05	1.9 ± 0.005	1.68 ± 0.006
*C. parapsilosis* (ATCC 22,019)	1.48 ± 0.005	1.61 ± 0.005	1.70 ± 0.005	1.90 ± 0.005	1.51 ± 0.01	1.2 ± 0.04	1.89 ± 0.005	1.68 ± 0.007
*C. glabrata* (ATCC 15,545)	1.5 ± 0.005	1.6 ± 0.005	1.71 ± 0.01	1.90 ± 0.005	1.5 ± 0.005	1.2 ± 0.05	1.9 ± 0.008	1.7 ± 0.005

The eight ANOVA tests along with accompanying post-hoc tests showed there were differences between control AgNPs (stock AgNP at standard pH and temperature) and the treated AgNPs (at different pH and temperatures). Despite of these differences in ZOI, AgNPs showed significant antifungal activities hence confirming their stability in all conditions of pH and temperatures.

### Fungal cell morphology and membrane integrity

**Fig. 12:**
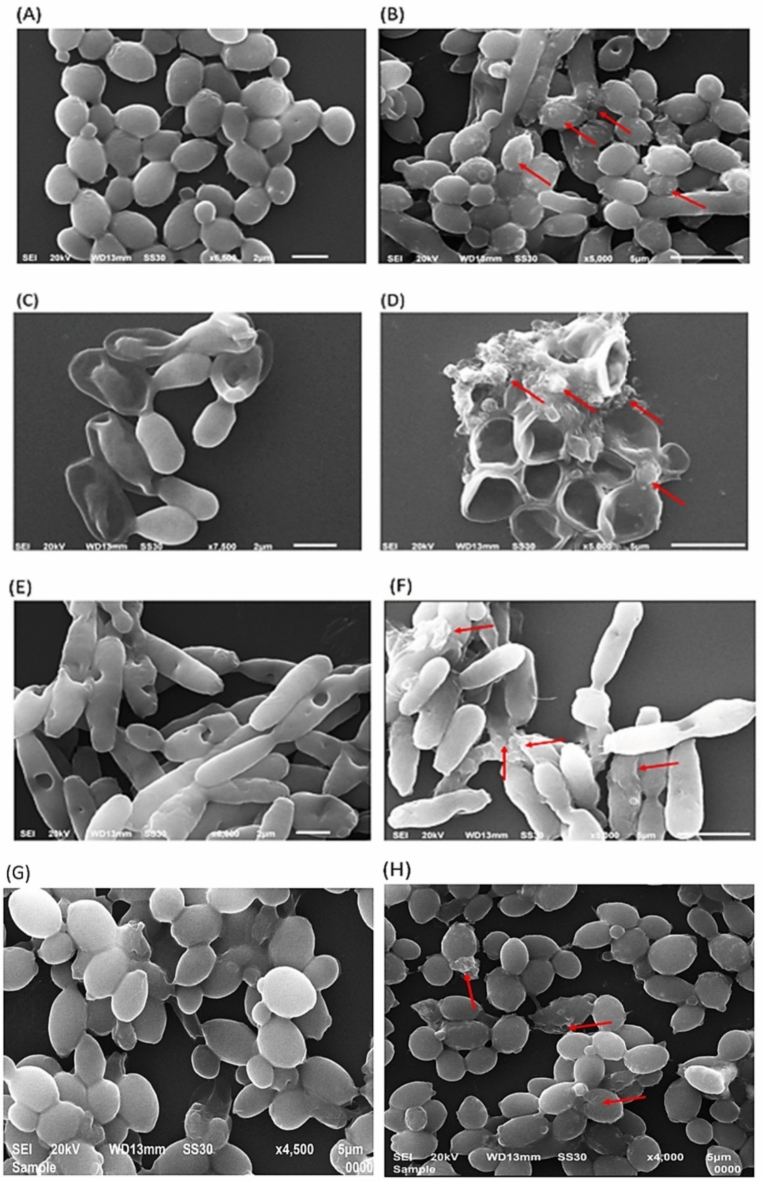
Scanning electron micrographs of *C. albicans*, *C. parapsilosis*, *C. krusei*, *C. glabrata* untreated cells (**A**,**C**,**E**,**G**) and treated with MIC value (0.003 ng/mL) of AgNPs (**B**,**D**,**F**,**H**).

The ultrastructural differences were visualized by SEM after treating different *Candida* spp. cells to AgNPs at respective MIC values (0.003 ng/mL). Cells treated with AgNPs showed cell shrinkage, disintegration, disorganized cell membrane, deformation and also leakage of cellular components, which further suggest that treatment of AgNPs can cause formation of pores in the fungal cell wall and cell membrane that leads to penetration of AgNPs inside the cell cytoplasm causing cell death^63^ (Fig. 12B,D,F,H). On the other hand, untreated control *Candida* cells exhibit smooth shape and intact morphology without any leakage of cellular components (Fig. 12A,C,E,G).

### Release of intracellular material

**Fig. 13:**
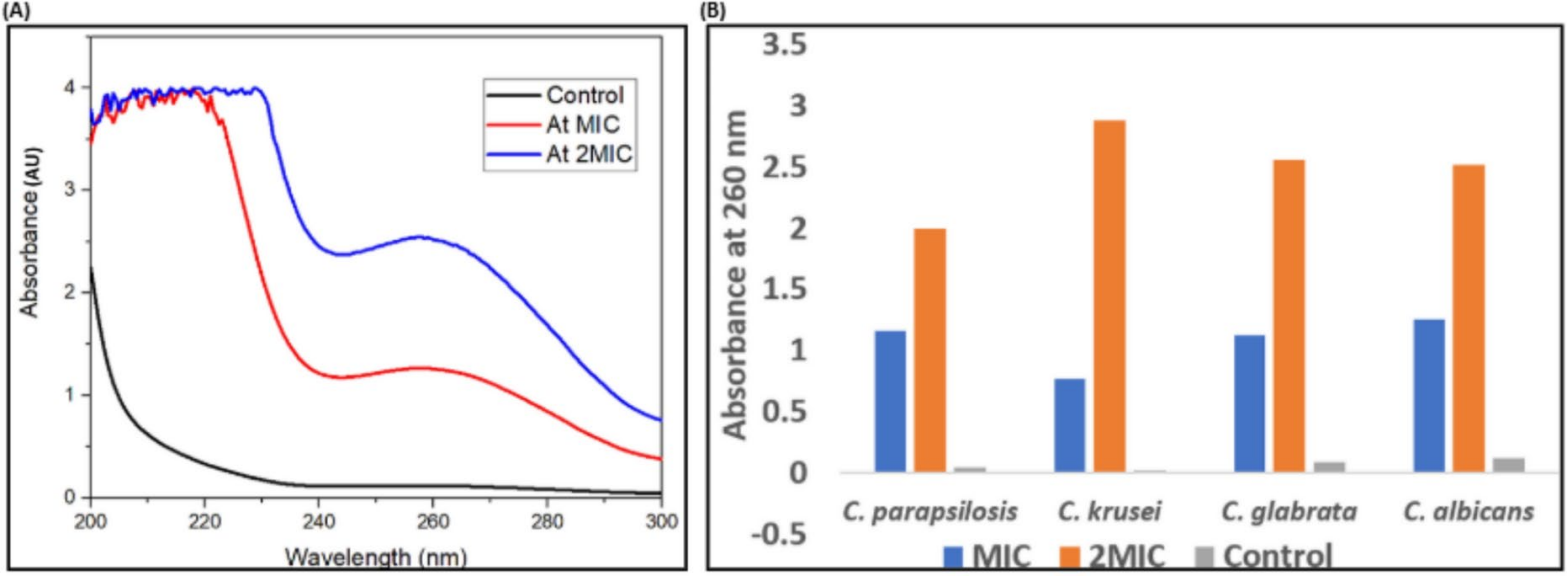
(**A**) Intracellular component release upon exposure of *C. albicans* to AgNPs (**B**) Concentration dependent effect of AgNPs (at MIC and 2MIC values) on different *Candida* spp. at 260 nm.

In this study, we have measured the effect of AgNPs on cell permeability and integrity of cell membranes. Spectrophotometric analysis of cell supernatant containing intracellular components such as nucleotides was done at wavelength 260 nm. *Candida* cells treated with AgNPs at MIC, 2MIC values (0.003 and 0.006 ng/mL respectively), exhibit increased absorbance at 260 nm due to the rupturing of cell membrane and release of intracellular components including DNA, purine and pyrimidine-based nucleotides. The effect was found to be concentration-dependent (2MIC > MIC) (Fig. 13A and B).

### Disruption of mitochondrial activity

**Fig. 14:**
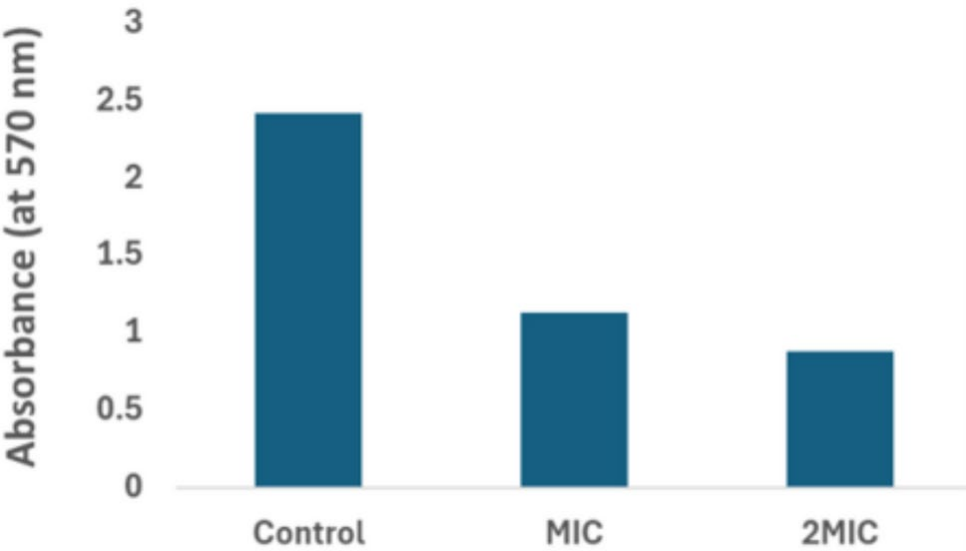
Effect of AgNPs (at MIC, 2MIC value) on mitochondrial activity of *C. albicans* demonstrated by bar graph and quantified by using MTT assay.

The mitochondria activity of *C. albicans* was determined through MTT assay. MTT is used as common indicator to check metabolic activity of cells. In healthy cells, yellow coloured MTT is reduced by mitochondrial enzymes (mitochondrial dehydrogenase) to purple coloured formazan. Treatment of *Candida* cells with AgNPs at MIC and 2MIC concentration (0.003 and 0.006 ng/mL respectively), has disrupted the mitochondrial activity in comparison to control *Candida* cells (Fig. 14).

### Ergosterol levels

**Fig. 15:**
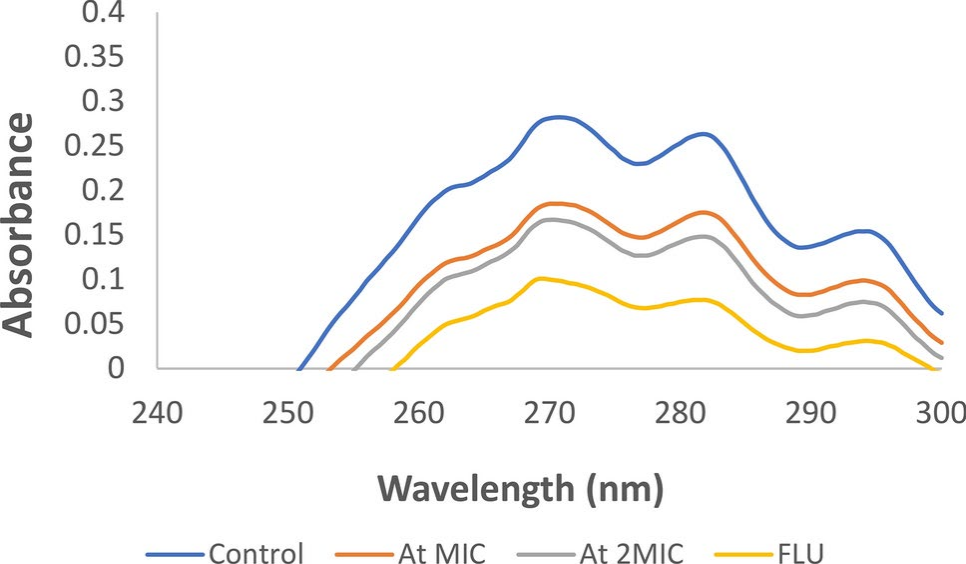
Spectroscopic Sterol analysis of *C. albicans* after 24 h incubation at varying concentration of AgNPs in YPD media. Spectral scanning of extracted sterols was done in UV range between (240–300 nm). FLU treated and untreated cells were used as controls.

The effect of AgNPs on the membrane of *C. albicans* cells was obtained by measuring ergosterol levels. AgNPs had significant concentration dependent inhibitory effect on ergosterol biosynthesis. The untreated control cells did not show any reduction in ergosterol levels while FLU treated and AgNPs treated *Candida* cells at their respective MIC and 2MIC values showed significant effects (Fig. 15). The effect is more in FLU treated cells as compared to AgNP treated cells as FLU has sole action on ergosterol synthetic pathway, while the mode of action of AgNPs on fungal cell may be multifaceted.

### Biofilm inhibition by AgNPs treatment

**Fig. 16:**
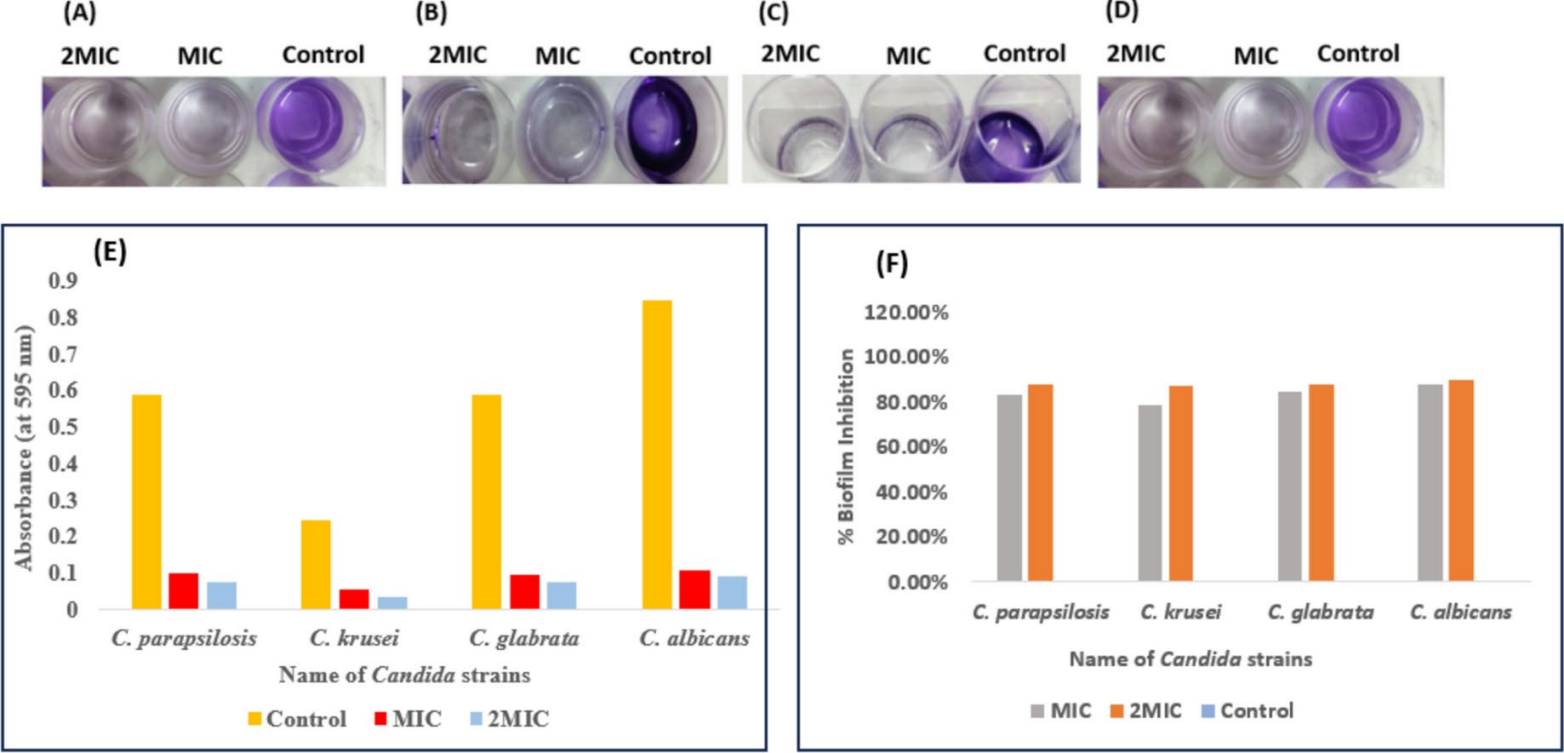
Biofilm inhibition by AgNPs assessed by crystal violet staining assay (**A**) *C. albicans* (**B**) *C. krusei* (**C**) *C. glabrata* (**D**) *C. parapsilosis* (**E**) Absorbance values at 595 nm of destaining solution from treated (MIC and 2MIC) and control samples (**F**) Biofilm inhibition (%) of *Candida* spp. upon treatment with AgNPs at respective MIC and 2MIC values.

Treatment and inhibition of *Candida* cells biofilm was analyzed by CV assay. In this study, we have tested AgNPs both at MIC, and 2MIC concentration that were able to inhibit the biofilm formation in all tested fungal strains **(**Fig. 16A–E). The various physicochemical factors of AgNPs including size, shape, surface area to volume ratio, capping and stabilizing agent on the surface of NPs determines their biological activity on target cells. All these parameters together affect the interactions between AgNPs and cell surface of living cells, adhesion of AgNPs on the cell surface, their uptake and penetration inside the cells.

It has been studied that the most virulent feature of *Candida* is to form biofilm that displays three-dimensional network of extracellular matrix (ECM) which leads to resistance against the drugs^64^. The biofilm matrix is composed of exopolysaccharides, nucleic acids (eDNA and eRNA), proteins, lipids and other biomolecules. ECM components promote adhesion of yeast cells to biotic and abiotic surfaces and forms water channels to allow entry of nutrients^44^. Mature biofilms show resistance to drug as they do not allow the entry of drugs to penetrate inside biofilms, and also results in chronic infections that are generally found to be resistant to commercially available antifungal drugs. Maximum colour will be seen (after destaining) in the control untreated cells as compared to AgNP treated cells. In this study, we have tested the ability of our AgNPs against matured or established biofilms of fungal strains. Treatment of 24 h mature *Candida* biofilm with green-synthesized AgNPs at their respective MIC and 2MIC values (0.003 and 0.006 ng/mL respectively), has led to inhibition of biofilm by 80–85% (Fig. 16F).

**Fig. 17:**
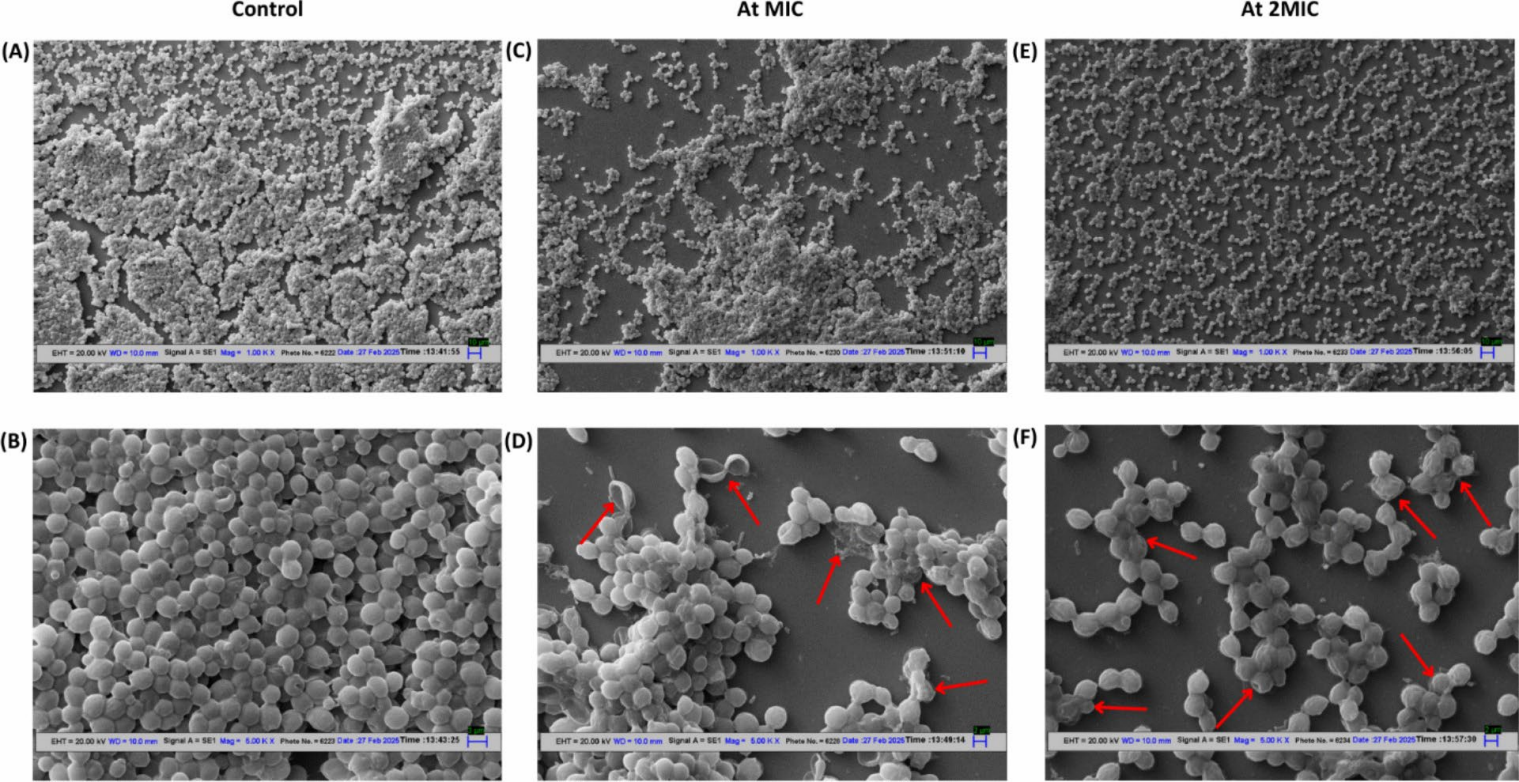
SEM observations of *C. albicans* biofilm (control, **A** and **B**) at different magnifications (1000 X, 5000 X), treated with MIC (**C**,**D**) and 2MIC (**E**,**F**) of AgNPs.

SEM images confirmed that, a biofilm reduction was observed when fungal cells are treated with green-synthesized AgNPs (at MIC and 2 MIC). Biofilms are defined as surface attached microbial communities that exhibit sessile and planktonic cells and are attached to underlying surface by the help of extracellular matrix^45^. These organized biofilm structure provides the resistance to yeast cells from environmental harsh conditions and protect them from host immune evasion systems. The findings obtained from this study, suggest that green synthesized AgNPs have the ability to inhibit biofilm formation. SEM analysis showed that AgNPs were able to substantially decrease cell density of biofilm associated cells (Fig. 17A,C,E). AgNPs inhibit biofilm formation by making changes in cell morphology including deep depressions on the surface of cell, and shrinkage of cell due to release of intracellular components (Fig. 17B,D,F) (indicated with red arrows). These changes observed in AgNPs treated biofilm cells can be linked to the changes in fungal cell membrane and cell wall components that can further decrease the pathogenicity and growth of fungal pathogens.

## Discussion

**Fig. 18:**
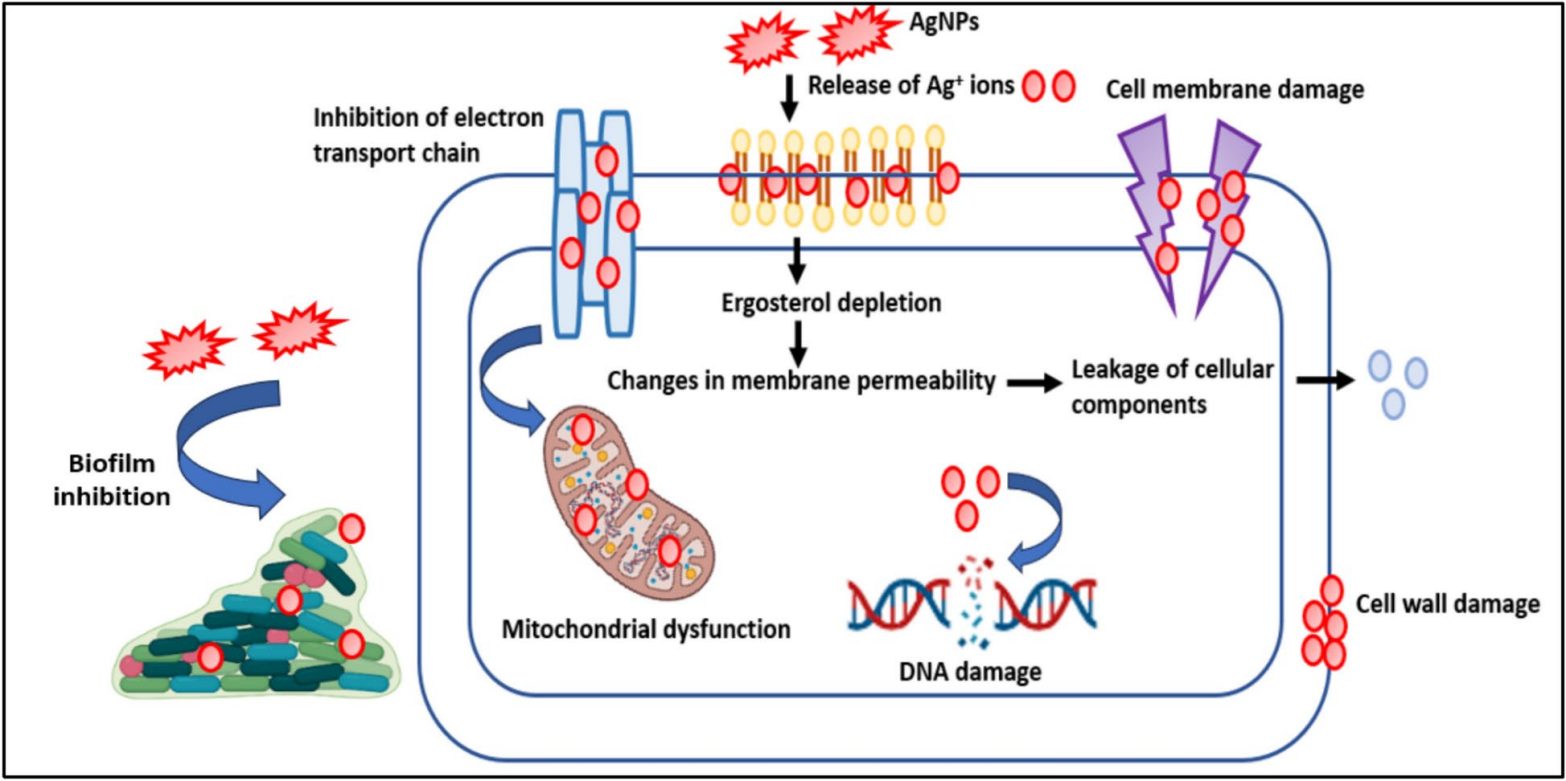
The detailed mechanism of green-synthesized AgNPs as potent antifungal agent against *Candida* spp.

Candidiasis due to *C. albicans* and *non-albicans Candida* spp. (NACS) has become a global threat in the healthcare sector due to the presence of very limited therapeutic drugs options. Thus, there is an urgent need to develop novel alternative drugs that are able to target multidrug-resistant fungal pathogens. The usage of NPs in the field of medicine can be considered as an innovative strategy to combat infectious diseases. The NPs synthesized from biological methods have controlled shape, size, stability and enhanced therapeutic activity as compared to those prepared from physical and chemical methods. AgNPs are widely employed as antinociceptive^65^, anticoagulant^66^, antihyperlipidemia agents^67^. The well-known anti-microbial activity of silver from ancient times has led to identification of AgNPs as antimicrobial agent. In this present study, we have identified the anticandidal activity of green-synthesized AgNPs obtained from the leaf extract of medicinal plant *S. bryopteris*. AgNPs demonstrated an interesting antifungal activity against four *Candida* spp. with an MIC and MFC values of 0.003 ng/mL and 0.006 ng/mL respectively. The various characteristic features of nanomaterial including shape, size, surface morphology, energy, charge, agglomeration state can affect their fate and biological interactions. Further, the surface chemistry, charge of NPs determines their agglomeration behaviour^68^. The NP surface-area-to-volume ratio is very important to be an effective anti-microbial drug and the presence of phytocompounds as coating agents around the surface of NPs determines their functionality. In this study, we have focussed on deciphering the mechanism of action of AgNP as an antifungal agent (Fig. 18). AgNPs were found to affect fungal cell morphology, disruption of cell membrane and cell wall of *Candida* spp. and cause dysfunction of mitochondrial activity along with the release of nuclear content. AgNPs also showed significant biofilm reduction activity against mature biofilms of *Candida* spp.

Recent study by Al-Otibi et al.^69^ has reported the antifungal activity of AgNPs synthesized from green tea leaves extract and has observed ultrastructural changes in *C. albicans* including irregular cell shape, size, pits on cell membrane and cell wall, leading to disruption of membrane and leakage of cellular components and ultimately cell death. Another study^60^ has reported the anti-candidal activity of AgNPs synthesized from fruit extract of *Punica granatum* against *C. albicans* (ATCC 18,804), *C. tropicalis* (ATCC 13,803) and *C. glabrata* (ATCC 15,545) and also observed synergistic effect of AgNP with commercially available itraconazole against these strains. Yet more studies^61^ have demonstrated the synthesis of AgNPs using aqueous leaf extract of *Origanum majorana* and evaluated their antibacterial activity against multidrug resistant bacterial pathogens. Although antimicrobial properties of AgNPs have been widely reported but very few studies have focused on the detailed mechanism behind these activities^33,70^. The proposed mechanism of antifungal action of AgNPs involves its direct effect on the fungal cell envelope including cell wall and membranes, leading to formation of pores in the cell membranes. These pores enable the entry of AgNPs into fungal cells leading to the denaturation of cellular biomolecules such as proteins along with DNA damage. Another mechanism that underlines the lethal effect of AgNPs against pathogenic fungi is their ability to promote the production of reactive oxygen species (ROS) that brings about immense oxidative stress followed by cell death^31^. The enhancement of antimicrobial drug activity can be achieved through their conjunction with metal NPs to overcome antimicrobial resistance^71^. There are several reports that indicate the role of Ag^+^ released from AgNPs for antimicrobial activity. Further, Ag^+^ reacts with the thiol and sulfhydryl group of the proteins and enzymes and leads to protein deactivation. In addition to this, it has also been observed that Ag^+^ intercalates between the purine and pyrimidine base pairs. The AgNPs also exhibit efflux pump inhibitory effects in two ways- firstly binding of AgNPs at active site of efflux pump prevents the extrusion of antimicrobial drugs from the cells and also disturbs the efflux pump kinetics^72^, and secondly AgNPs alters the proton gradient leading to disruption of membrane potential that plays crucial role in the maintenance of efflux pump activity. The usage of other metallic NPs such as zinc oxide NPs synthesized from pomegranate peel extract, was found to exhibit antifungal activity against fungal strains such as *C. albicans*, *C. glabrata*, *C. tropicalis*^54^. The synthesis of zinc oxide NPs from the leaf extract of *Camellia sinensis* (green tea) has also been tested along with the commercially available antifungal drug such as nystatin and terbinafine against *C. albicans* (ATCC 29,213), *C. glabrata* (ATCC 25,922), and *C. tropicalis* (ATCC 33,592) by disc diffusion method^73^.

The green synthesis of AgNPs has major applications in medicinal field including cancer therapy, drug delivery, bioimaging due to absence of toxic and hazardous chemicals in it. Apart from all these properties, NPs are utilized as nanocarriers as they exhibit enhanced half-life and stability of drug carrier in circulation and provide target-specific controlled release of drugs. Conjugating AgNPs with peptide drugs can be an interesting approach for the treatment of various diseases. A study by Majeed et al.^48^ has reported the conjugation of TAT-peptide with green-synthesized AgNPs from *Staphylococcus aureus* cell free extract and tested against breast adenocarcinoma. For the drug delivery purposes of active ingredient, nano-emulsions are prepared to enhance the bio-availability and adsorption of essential oil derived phytocompounds^74^. Apart from this, the green synthesis of zinc oxide NPs from the extract of *Streptomyces barrnensis* and its active metabolite called as ka 9-Ethyl-1,4,6,9,10-pentahydroxy-7,8,9,10-tetrahydrotetracene-5,12-dione is loaded into nano-emulsion to improve their solubility and bioavailability, and has been further evaluated for its antibacterial activity against bacterial pathogens^75^. Moreover, the mechanism of biofilm inhibition is related to the damage to the exopolysaccharides layers and protein components that serve as extracellular matrix (ECM). The ECM of biofilm contains polysaccharides such as β-1,3 glucan, β-1,6 glucan, α-1,6 mannan, α-1,2-branched mannan that further leads to emergence of antifungal drug resistance in *Candida* biofilms. The mannan and glucan components of ECM forms a net like structure that sequesters the antifungal drugs via non-covalent interactions and do not allow the entry inside biofilm cells. The damage of ECM components by NPs can further result into the detachment of biofilm cells and nutrient depletion resulting into fungal cell death. In yet another study the use of gold nanoparticles in conjugation with Taxol from fungal cells was used against bacterial infections and as anti-cancer agents^76^. Green synthesized AgNPs in conjunction with fosfomycin displayed bactericidal activity against nosocomial bacterial pathogens^77^

The nanoparticles can therefore have immense applications as they can be used as anti-cancer, anti-coagulant, anti-bacterial, anti-fungal agents. They can be employed as nano-carriers for effective and targeted drug deliver and can effectively work in conjugation with known drugs to increase their efficacy. The challenge is therefore to make these NPs biocompatible and with least toxic side effects. The present study is therefore one such endeavour that has led to green synthesis of AgNPs with very high efficacy against drug resistant *Candida* strains.

## Conclusion

The study presents a sustainable and ecofriendly approach to synthesize AgNPs using aqueous leaf extract of *S. bryopteris*. This method of synthesis provides monodispersed AgNPs, having negative zeta potential, spherical morphology and have the ability to target human fungal pathogens. Further to know the detailed efficacy and the molecular basis of their action preventing fungal biofilm formation and inhibiting other virulence factors in the pathogenesis of *Candida*, additional studies are required. The findings obtained in this study can make a good contribution in the field of nanomedicine to provide novel insights for the development of effective and therapeutic antifungal drug against drug resistant *Candida* spp.

## Data Availability

The datasets generated and/or analysed during the current study are available from the corresponding author on reasonable request.

## References

[1] Thakkar, K. N., Mhatre, S. S. & Parikh, R. Y. Biological synthesis of metallic nanoparticles. *Nanomed. Nanotechnol. Biol. Med.* **6**(2), 257–262 (2010). 10.1016/j.nano.2009.07.002 19616126

[2] Rozhin, A. et al. Biogenic silver nanoparticles: Synthesis and application as antibacterial and antifungal agents. *Micromachines* **12**(12), 1480 (2021). 34945330 10.3390/mi12121480 PMC8708042

[3] Khoshnevisan, K. et al. Nanomaterial based electrochemical sensing of the biomarker serotonin: A comprehensive review. *Microchim. Acta* **186**, 1–21 (2019). 10.1007/s00604-018-3069-y 30610391

[4] Ingale, A. G. & Chaudhari, A. N. Biogenic synthesis of nanoparticles and potential applications: an eco-friendly approach. *J. Nanomed. Nanotechol.* **4**(165), 1–7 (2013).

[5] Tripathi, D. K. et al. An overview on manufactured nanoparticles in plants: Uptake, translocation, accumulation and phytotoxicity. *Plant Physiol. Biochem.* **110**, 2–12 (2017). 27601425 10.1016/j.plaphy.2016.07.030

[6] Rather, G. A. et al. Biosynthesis of Zinc oxide nanoparticles using Bergenia ciliate aqueous extract and evaluation of their photocatalytic and antioxidant potential. *Inorg. Chem. Commun.* **134**, 109020 (2021).

[7] Ghomi, A. R. G. et al. Fungus-mediated extracellular biosynthesis and characterization of zirconium nanoparticles using standard penicillium species and their preliminary bactericidal potential: a novel biological approach to nanoparticle synthesis. *Iran. J. Pharm. Res. IJPR* **18**(4), 2101 (2019). 32184873 10.22037/ijpr.2019.112382.13722 PMC7059062

[8] Vahidi, H., Kobarfard, F., Alizadeh, A., Saravanan, M. & Barabadi, H. Green nanotechnology-based tellurium nanoparticles: Exploration of their antioxidant, antibacterial, antifungal and cytotoxic potentials against cancerous and normal cells compared to potassium tellurite. *Inorg. Chem. Commun.* **124**, 108385 (2021).

[9] Vahidi, H. et al. Mycosynthesis and characterization of selenium nanoparticles using standard penicillium chrysogenum PTCC 5031 and their antibacterial activity: A novel approach in microbial nanotechnology. *Nanomed. J.* **7**(4), 315–323 (2020).

[10] Khan, I. et al. The effect of biogenic manufactured silver nanoparticles on human endothelial cells and zebrafish model. *Sci. Total Environ.* **679**, 365–377 (2019). 31085416 10.1016/j.scitotenv.2019.05.045

[11] Kaur, H., & Wadhwa, K. Exploration of new plant-based nanoparticles with potential antifungal activity and their mode of action. In *Advances in Antifungal Drug Development: Natural Products with Antifungal Potential*, 345–371 (Springer Nature Singapore, 2024).

[12] Noorbazargan, H. et al. Anti-cancer & anti-metastasis properties of bioorganic-capped silver nanoparticles fabricated from Juniperus chinensis extract against lung cancer cells. *AMB Express* **11**(1), 61 (2021). 33900505 10.1186/s13568-021-01216-6 PMC8076435

[13] Yadav, V., Kapoor, N. & Ghorai, S. M. Green synthesis of silver nanoparticles from aqueous leaf extract of *Selaginella bryopteris*. *Curr. Bioact. Compd.* **16**(4), 449–459 (2020).

[14] Pandey, V. et al. Desiccation-induced physiological and biochemical changes in resurrection plant, *Selaginella**bryopteris*. *J. Plant Physiol.* **167**(16), 1351–1359 (2010). 20605652 10.1016/j.jplph.2010.05.001

[15] Adnan, M. et al. Phytochemistry, bioactivities, pharmacokinetics and toxicity prediction of Selaginella repanda with its anticancer potential against human lung, breast and colorectal carcinoma cell lines. *Molecules* **26**(3), 768 (2021). 33540783 10.3390/molecules26030768 PMC7867377

[16] Muema, F. W., Liu, Y., Zhang, Y., Chen, G. & Guo, M. Flavonoids from *Selaginella**doederleinii* Hieron and their antioxidant and antiproliferative activities. *Antioxidants* **11**(6), 1189 (2022). 35740086 10.3390/antiox11061189 PMC9229023

[17] Dai, Y., But, P. P. H., Chu, L. M. & Chan, Y. P. Inhibitory effects of *Selaginella**tamariscina* on immediate allergic reactions. *Am. J. Chin. Med.* **33**(06), 957–966 (2005). 16355452 10.1142/S0192415X05003442

[18] Kunert, O. et al. Antiplasmodial and leishmanicidal activity of biflavonoids from Indian *Selaginella**bryopteris*. *Phytochem. Lett.* **1**(4), 171–174 (2008).

[19] Yassin, M. T., Mostafa, A. A. & Al-Askar, A. A. Anticandidal and anti-carcinogenic activities of *Mentha**longifolia* (Wild Mint) extracts in vitro. *J. King Saud Univ. Sci.* **32**(3), 2046–2052 (2020).

[20] Yassin, M. T., Mostafa, A. A. F. & Al-Askar, A. A. In vitro anticandidal potency of *Syzygium**aromaticum* (clove) extracts against vaginal candidiasis. *BMC Complement. Med. Ther.* **20**, 1–9 (2020). 32020877 10.1186/s12906-020-2818-8 PMC7076834

[21] Wadhwa, K. et al. A systematic review on antimicrobial activities of green synthesized Selaginella silver nanoparticles. *Expert Rev. Mol. Med.*10.1017/erm.2023.21 (2023). 37534437 10.1017/erm.2023.21 PMC10752228

[22] Wadhwa, K., Kaur, H., Kapoor, N. & Brogi, S. Identification of sesamin from sesamum indicum as a potent antifungal agent using an integrated in silico and biological screening platform. *Molecules* **28**(12), 4658 (2023). 37375219 10.3390/molecules28124658 PMC10304600

[23] Xiao, Z., Wang, Q., Zhu, F. & An, Y. Epidemiology, species distribution, antifungal susceptibility and mortality risk factors of candidemia among critically ill patients: A retrospective study from 2011 to 2017 in a teaching hospital in China. *Antimicrob. Resist. Infect. Control* **8**, 1–7 (2019). 31161036 10.1186/s13756-019-0534-2 PMC6542075

[24] Hossain, C. M. et al. Antifungals and drug resistance. *Encyclopedia* **2**(4), 1722–1737 (2022).

[25] Sawant, B. & Khan, T. Recent advances in delivery of antifungal agents for therapeutic management of candidiasis. *Biomed. Pharmacother.* **96**, 1478–1490 (2017). 29223551 10.1016/j.biopha.2017.11.127

[26] Tian, J. et al. Topical delivery of silver nanoparticles promotes wound healing. *ChemMedChem Chem. Enabl. Drug Discov.* **2**(1), 129–136 (2007). 10.1002/cmdc.200600171 17075952

[27] Mazurak, V. C., Burrell, R. E., Tredget, E. E., Clandinin, M. T. & Field, C. J. The effect of treating infected skin grafts with Acticoat™ on immune cells. *Burns* **33**(1), 52–58 (2007). 17079089 10.1016/j.burns.2006.04.027

[28] Bobo, D., Robinson, K. J., Islam, J., Thurecht, K. J. & Corrie, S. R. Nanoparticle-based medicines: A review of FDA-approved materials and clinical trials to date. *Pharm. Res.* **33**, 2373–2387 (2016). 27299311 10.1007/s11095-016-1958-5

[29] Boateng, J. & Catanzano, O. Advanced therapeutic dressings for effective wound healing—a review. *J. Pharm. Sci.* **104**(11), 3653–3680 (2015). 26308473 10.1002/jps.24610

[30] Inam, M. et al. Size and shape affect the antimicrobial activity of quaternized nanoparticles. *J. Polym. Sci. Part A Polym. Chem.* **57**(3), 255–259 (2019).

[31] Hamida, R. S., Ali, M. A., Goda, D. A., Khalil, M. I. & Al-Zaban, M. I. Novel biogenic silver nanoparticle-induced reactive oxygen species inhibit the biofilm formation and virulence activities of methicillin-resistant *Staphylococcus**aureus* (MRSA) strain. *Front. Bioeng. Biotechnol.* **8**, 433 (2020). 32548095 10.3389/fbioe.2020.00433 PMC7270459

[32] Jebril, S., Fdhila, A. & Dridi, C. Nanoengineering of eco-friendly silver nanoparticles using five different plant extracts and development of cost-effective phenol nanosensor. *Sci. Rep.* **11**(1), 22060 (2021). 34764386 10.1038/s41598-021-01609-4 PMC8586347

[33] Hamida, R. S., Ali, M. A., Goda, D. A. & Redhwan, A. Anticandidal potential of two cyanobacteria-synthesized silver nanoparticles: Effects on growth, cell morphology, and key virulence attributes of Candida albicans. *Pharmaceutics* **13**(10), 1688 (2021). 34683981 10.3390/pharmaceutics13101688 PMC8539685

[34] Muhammad Tahir, H. et al. Synthesis of sericin-conjugated silver nanoparticles and their potential antimicrobial activity. *J. Basic Microbiol.* **60**(5), 458–467 (2020). 32115731 10.1002/jobm.201900567

[35] Summer, M. et al. Bactericidal potential of different size sericin-capped silver nanoparticles synthesized by heat, light, and sonication. *J. Basic Microbiol.* **63**(9), 1016–1029 (2023). 36879387 10.1002/jobm.202200632

[36] Mumtaz, S. et al. Analysis of the antimicrobial potential of sericin-coated silver nanoparticles against human pathogens. *Microsc. Res. Tech.* **86**(3), 320–330 (2023). 36582143 10.1002/jemt.24273

[37] CLSI (Clinical and Laboratory Standards Institute). Reference Method for Broth Dilution Antifungal Susceptibility Testing of Yeasts; Fourth Informational Supplement M27-S4 (Clinical and Laboratory Standard Institute, 2012).

[38] Marques, M. B., Brookings, E. S., Moser, S. A., Sonke, P. B. & Waites, K. B. Comparative in vitro antimicrobial susceptibilities of nosocomial isolates of Acinetobacter baumannii and synergistic activities of nine antimicrobial combinations. *Antimicrob. Agents Chemother.* **41**(5), 881–885 (1997). 9145838 10.1128/aac.41.5.881 PMC163819

[39] Lyu, Y. et al. Antimicrobial activity, improved cell selectivity and mode of action of short PMAP-36-derived peptides against bacteria and *Candida*. *Sci. Rep.* **6**, 27258 (2016). 27251456 10.1038/srep27258 PMC4890124

[40] Shahina, Z., Ndlovu, E., Persaud, O., Sultana, T. & Dahms, T. E. *Candida**albicans* reactive oxygen species (ROS)-dependent lethality and ROS-independent hyphal and biofilm inhibition by eugenol and citral. *Microbiol. Spectr.* **10**(6), e03183-e3222 (2022). 36394350 10.1128/spectrum.03183-22 PMC9769929

[41] Ansari, M. A., Fatima, Z. & Hameed, S. Anticandidal effect and mechanisms of monoterpenoid, perillyl alcohol against *Candida**albicans*. *PLoS One* **11**(9), e0162465 (2016). 27627759 10.1371/journal.pone.0162465 PMC5023166

[42] Arthington-Skaggs, B. A., Jradi, H., Desai, T. & Morrison, C. J. Quantitation of ergosterol content: Novel method for determination of fluconazole susceptibility of Candida albicans. *J. Clin. Microbiol.* **37**(10), 3332–3337 (1999). 10488201 10.1128/jcm.37.10.3332-3337.1999 PMC85559

[43] Pierce, C. G. et al. A simple and reproducible 96-well plate-based method for the formation of fungal biofilms and its application to antifungal susceptibility testing. *Nat. Protoc.* **3**(9), 1494–1500 (2008). 18772877 10.1038/nport.2008.141 PMC2741160

[44] Rodrigues, C. F. et al. The effectiveness of voriconazole in therapy of *Candida glabrata’s* biofilms oral infections and its influence on the matrix composition and gene expression. *Mycopathologia* **182**, 653–664 (2017). 28439794 10.1007/s11046-017-0135-7

[45] Rodrigues, C. F., Rodrigues, M. E. & Henriques, M. Susceptibility of *Candida glabrata* biofilms to echinocandins: Alterations in the matrix composition. *Biofouling* **34**(5), 569–578 (2018). 29798695 10.1080/08927014.2018.1472244

[46] Pal, L. C., Gautam, A., Pande, V. & Rao, C. V. Anticancer property of *Selaginella**bryopteris* (L.) Bak. against hepatocellular carcinoma in vitro and in vivo. *Phytomed. Plus* **2**(1), 100201 (2022).

[47] Gautam, A., Pal, L. C., Rao, C. V., & Kumar, V. The Role of Indian Magical Herb Selaginella bryopteris L. (Selaginaceae) In Pharmacotherapeutic Perspective: An Overview. *Pharmacogn. J.*, *15*(1) (2023).

[48] Majeed, S. et al. Bioengineering of green-synthesized TAT peptide-functionalized silver nanoparticles for apoptotic cell-death mediated therapy of breast adenocarcinoma. *Talanta* **253**, 124026 (2023).

[49] Mallmann, E. J. J. et al. Antifungal activity of silver nanoparticles obtained by green synthesis. *Rev. Inst. Med. Trop. Sao Paulo* **57**(2), 165–167 (2015). 25923897 10.1590/S0036-46652015000200011 PMC4435016

[50] Ayromlou, A., Masoudi, S. & Mirzaie, A. Scorzonera calyculata aerial part extract mediated synthesis of silver nanoparticles: Evaluation of their antibacterial, antioxidant and anticancer activities. *J. Cluster Sci.* **30**, 1037–1050 (2019).

[51] Car, J. & Krstulović, N. Analytical model for determination of size-distribution of colloidal silver nanoparticles from surface plasmon resonance wavelength and dielectric functions. *Nanomaterials* **12**(19), 3474 (2022). 36234602 10.3390/nano12193474 PMC9565655

[52] Bamsaoud, S. F., Basuliman, M. M., Bin-Hameed, E. A., Balakhm, S. M. & Alkalali, A. S. The effect of volume and concentration of AgNO3 aqueous solutions on silver nanoparticles synthesized using Ziziphus Spina-Christi leaf extract and their antibacterial activity. *J. Phys. Conf. Ser.* **1900**(1), 012005 (2021).

[53] Jain, S. & Mehata, M. S. Medicinal plant leaf extract and pure flavonoid mediated green synthesis of silver nanoparticles and their enhanced antibacterial property. *Sci. Rep.* **7**(1), 15867 (2017). 29158537 10.1038/s41598-017-15724-8 PMC5696514

[54] Yassin, M. T., Mostafa, A. A. F., Al-Askar, A. A. & Al-Otibi, F. O. Facile green synthesis of zinc oxide nanoparticles with potential synergistic activity with common antifungal agents against multidrug-resistant candidal strains. *Crystals* **12**(6), 774 (2022).

[55] Szerencsés, B. et al. Size-dependent activity of silver nanoparticles on the morphological switch and biofilm formation of opportunistic pathogenic yeasts. *BMC Microbiol.* **20**, 1–13 (2020). 32571216 10.1186/s12866-020-01858-9 PMC7309973

[56] Mohammad, N. H. et al. Gamma-ray and sunlight-induced synthesis of silver nanoparticles using bacterial cellulose and cell-free filtrate produced by Komagataeibacter rhaeticus N1 MW322708 strain. *Cellulose* **29**(3), 1791–1805 (2022).

[57] Mirzaie, A. et al. Phyto-fabrication of silver nanoparticles using typha azerbaijanensis aerial part and root extracts. *Iran. J. Public Health* **51**(5), 1097 (2022). 36407723 10.18502/ijph.v51i5.9425 PMC9643224

[58] Gevorgyan, S. et al. Structural characterization and antibacterial activity of silver nanoparticles synthesized using a low-molecular-weight Royal Jelly extract. *Sci. Rep.* **12**(1), 14077 (2022). 35982108 10.1038/s41598-022-17929-y PMC9388513

[59] Yassin, M. T., Mostafa, A. A. F., Al-Askar, A. A. & Al-Otibi, F. O. Synergistic antibacterial activity of green synthesized silver nanomaterials with colistin antibiotic against multidrug-resistant bacterial pathogens. *Crystals* **12**(8), 1057 (2022).

[60] Yassin, M. T., Mostafa, A. A. F., Al-Askar, A. A. & Al-Otibi, F. O. Synergistic antifungal efficiency of biogenic silver nanoparticles with itraconazole against multidrug-resistant candidal strains. *Crystals* **12**(6), 816 (2022).

[61] Yassin, M. T., Mostafa, A. A. F., Al-Askar, A. A. & Al-Otibi, F. O. Facile green synthesis of silver nanoparticles using aqueous leaf extract of *Origanum**majorana* with potential bioactivity against multidrug resistant bacterial strains. *Crystals* **12**(5), 603 (2022).

[62] Yassin, M. T., Al-Askar, A. A., Maniah, K. & Al-Otibi, F. O. Green synthesis of zinc oxide nanocrystals utilizing *Origanum**majorana* leaf extract and their synergistic patterns with colistin against multidrug-resistant bacterial strains. *Crystals* **12**(11), 1513 (2022).

[63] Jain, K. et al. Antifungal potency of biosynthesized silver nanoparticles derived from marine diatoms against multidrug-resistant *Candida auris* and *Pichia kudriavzevii*. *ChemistrySelect* **10**(3), e202404088 (2025).

[64] Silva, S., Rodrigues, C. F., Araújo, D., Rodrigues, M. E. & Henriques, M. *Candida* species biofilms’ antifungal resistance. *J. Fungi* **3**(1), 8 (2017). 10.3390/jof3010008 PMC5715972 29371527

[65] Barabadi, H. et al. Nanobiotechnological approaches in antinociceptive therapy: animal-based evidence for analgesic nanotherapeutics of bioengineered silver and gold nanomaterials. *Adv. Coll. Interface. Sci.* **316**, 102917 (2023). 10.1016/j.cis.2023.102917 37150042

[66] Barabadi, H. et al. Nanobiotechnological approaches in anticoagulant therapy: The role of bioengineered silver and gold nanomaterials. *Talanta* **256**, 124279 (2023). 36709710 10.1016/j.talanta.2023.124279

[67] Barabadi, H. et al. Animal-based evidence supports the influence of biogenic silver and gold nanomaterials on the serum lipid profile: a novel approach in antihyperlipidemia management. *Results Surf. Interfaces* **16**, 100264 (2024).

[68] Gao, X. & Lowry, G. V. Progress towards standardized and validated characterizations for measuring physicochemical properties of manufactured nanomaterials relevant to nano health and safety risks. *NanoImpact* **9**, 14–30 (2018).

[69] Al-Otibi, F. O., Yassin, M. T., Al-Askar, A. A. & Maniah, K. Green biofabrication of silver nanoparticles of potential synergistic activity with antibacterial and antifungal agents against some nosocomial pathogens. *Microorganisms* **11**(4), 945 (2023). 37110368 10.3390/microorganisms11040945 PMC10144991

[70] Radhakrishnan, V. S. et al. Silver nanoparticles induced alterations in multiple cellular targets, which are critical for drug susceptibilities and pathogenicity in fungal pathogen (Candida albicans). *Int. J. Nanomed.* **13**, 2647 (2018). 10.2147/IJN.S150648 PMC5937493 29760548

[71] El-Sherbiny, G. M. et al. Biogenic synthesis of CuO-NPs as nanotherapeutics approaches to overcome multidrug-resistant *Staphylococcus**aureus* (MDRSA). *Artif. Cells Nanomed. Biotechnol.* **50**(1), 260–274 (2022). 36191138 10.1080/21691401.2022.2126492

[72] Behdad, R. et al. Efflux pump inhibitory activity of biologically synthesized silver nanoparticles against multidrug-resistant Acinetobacter baumannii clinical isolates. *J. Basic Microbiol.* **60**(6), 494–507 (2020). 32301139 10.1002/jobm.201900712

[73] Yassin, M. T. et al. Synergistic anticandidal activities of greenly synthesized ZnO nanomaterials with commercial antifungal agents against candidal infections. *Micromachines* **14**(1), 209 (2023). 36677271 10.3390/mi14010209 PMC9865458

[74] El-Sherbiny, et al. Nanoemulsion of cinnamon oil to combat colistin-resistant Klebsiella pneumoniae and cancer cells. *Microb. Pathog.* **192**, 106705 (2024). 38761892 10.1016/j.micpath.2024.106705

[75] Kalaba, M. H., El-Sherbiny, G. M., Ewais, E. A., Darwesh, O. M. & Moghannem, S. A. Green synthesis of zinc oxide nanoparticles (ZnO-NPs) by Streptomyces baarnensis and its active metabolite (Ka): A promising combination against multidrug-resistant ESKAPE pathogens and cytotoxicity. *BMC Microbiol.* **24**(1), 254 (2024). 38982372 10.1186/s12866-024-03392-4 PMC11232237

[76] Abdel-Fatah, S. S. et al. Boosting the anticancer activity of *Aspergillus**flavus* “endophyte of Jojoba” taxol via conjugation with gold nanoparticles mediated by γ-irradiation. *Appl. Biochem. Biotechnol.* **194**, 3558–3581 (2022). 35438406 10.1007/s12010-022-03906-8 PMC9270289

[77] Aljeldah, M. M., Yassin, M. T., Mostafa, A. A. F. & Aboul-Soud, M. A. M. Synergistic antibacterial potential of greenly synthesized silver nanoparticles with Fosfomycin against some nosocomial bacterial pathogens. *Infect Drug Resist.* **16**, 125–142 (2023). 36636381 10.2147/IDR.S394600 PMC9831080

